# Machine‐Learning‐Assisted Impedance Component Analysis Enables Standardizable Surface Protein Analysis of Extracellular Vesicles Using Engineered Nanovesicles

**DOI:** 10.1002/advs.77452

**Published:** 2026-08-29

**Authors:** Jaeyoon Song, Neethu Ramakrishnan, Sehyeon Kim, Huiseop Lee, Youngeun Kwon, Jinsik Kim

**Affiliations:** ^1^ Department of Biomedical Engineering, College of Life Science and Biotechnology Dongguk University Seoul Republic of Korea

**Keywords:** amyloid beta, engineered nanovesicles, extracellular vesicles, impedance spectroscopy, machine learning

## Abstract

Characterizing surface protein heterogeneity on extracellular vesicles remains challenging but essential for understanding their biological functions and clinical applications. Here, this study introduces an integrated platform that combines engineered cell‐derived nanovesicles, used as model standards with controlled surface protein states, and machine learning‐optimized impedance spectroscopy. Cell‐derived nanovesicles with defined surface protein densities are generated by extruding HeLa cells expressing 1, 3, or 9 copies of amyloid‐β 42, establishing a series of reference vesicles with precisely controlled oligomeric configurations. Through systematic evaluation of impedance features across frequencies from 10 Hz to 1 MHz, machine learning identifies reactance changes at 1 kHz as optimal for distinguishing oligomeric states on the vesicle membrane. Equivalent‐circuit modeling reveals that membrane capacitance correlates with protein oligomerization, and structural predictions explain the mechanistic basis. The platform also enables label‐free, time‐resolved analysis of Aβ oligomer formation directly on vesicular membranes, providing insights into aggregation dynamics. This platform establishes standardizable reference materials using engineered nanovesicles and a quantitative framework for extracellular vesicle surface protein analysis, offering a broadly applicable method for studying membrane‐associated protein dynamics relevant to neurodegenerative diseases and advancing extracellular vesicle‐based diagnostics.

## Graphical abstract

This study establishes a universally applicable framework for vesicle surface analysis by combining engineered amyloid‐β‐displaying nanovesicles with machine‐learning‐optimized impedance spectroscopy. Equivalent‐circuit modeling reveals that membrane capacitance correlates with the surface protein states, enabling label‐free quantification of vesicle surface heterogeneity. This approach provides foundational principles for developing clinically relevant, standardizable EV surface analysis platforms applicable beyond Aβ to diverse surface markers.

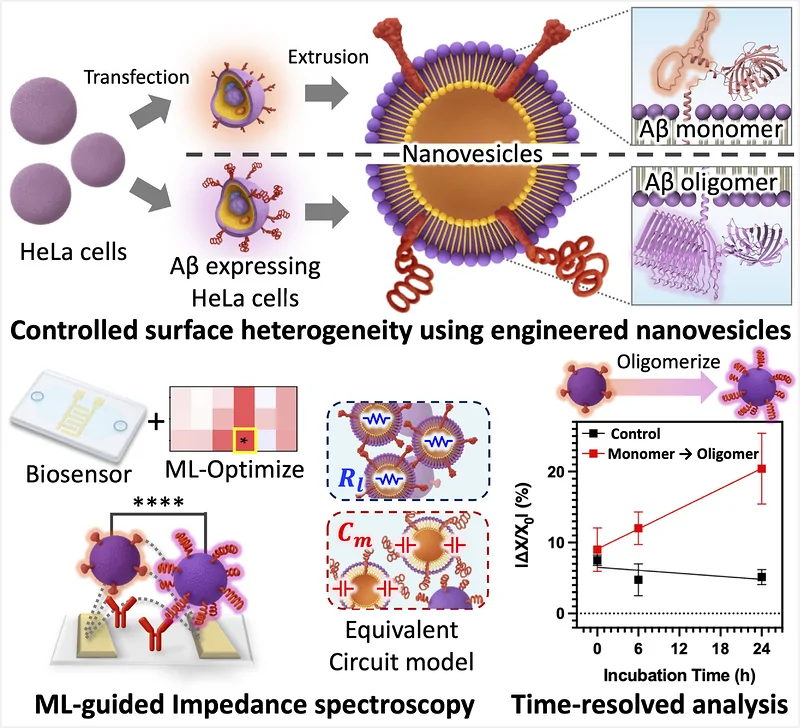

## 1 Introduction

Impedance spectroscopy is a versatile electrical characterization technique that enables detailed analysis of the physical properties and structural composition of various systems [1]. By applying small alternating current signals over a broad frequency range, this method probes the electrochemical responses of system components, providing label‐free and intact information about their composition and structure. These features have led to its widespread use in fields such as batteries, solar cells, and polymer science, and have also driven its growing adoption in biosensor development. Impedance‐based (impedimetric) biosensors, which detect changes in electrical impedance upon analyte binding, have been successfully developed for a range of targets, including hormones, proteins, and cells [2].

Impedance‐based analysis of extracellular vesicles (EVs) has emerged as a promising approach to addressing fundamental challenges in EV characterization. EVs are lipid bilayer–enclosed nanoparticles (30–1000 nm) that mediate intercellular communication, and their surface proteins play crucial roles in targeting specificity and functional responses [3, 4]. Both quantitative analysis of EV abundance and functional characterization of cargo distribution, in the lumen and on the membrane, are essential for understanding EV biology and enabling clinical applications [5]. Impedance analysis offers unique advantages for meeting these requirements: its label‐free nature and capacity to probe system components intact align well with the analytical demands of EV research. Recent impedance‐based EV studies have demonstrated capabilities beyond simple quantitative detection [6, 7], including comparative analysis of membrane protein expression using various antibodies [8]. Furthermore, analogous to single‐cell impedance analysis, where different measurement frequencies provide information about membrane and interior (lumenal) properties [9, 10, 11], frequency‐dependent impedance measurements of EVs can yield information about both compartments, enabling size determination, membrane protein analysis, and EV profiling for classification by cellular origin [12, 13, 14]. Notably, recent advances have enabled single‐EV‐level analysis, with real‐time monitoring of vesicle size and surface antibody binding, using impedance spectra as “fingerprints” for EV type classification [15]. These capabilities position impedance spectroscopy as a compelling alternative to established EV analysis methods. Unlike lysis‐dependent techniques such as RT–qPCR [16, 17, 18], next‐generation sequencing (NGS) [19, 20], and immunoblotting or ELISA [21, 22, 23], which disrupt vesicle membranes and alter the native arrangement of surface proteins, impedance analysis preserves vesicle integrity. Moreover, impedance spectroscopy shares the spectral analysis capabilities of other promising label‐free methods, such as surface‐enhanced Raman scattering (SERS) [24, 25, 26] and surface plasmon resonance (SPR) [27, 28, 29], enabling characterization of both lumenal biomolecules and surface proteins without labeling.

Despite the demonstrated potential of impedance‐based EV analysis, several critical barriers have limited its widespread adoption compared with established methods. The first challenge lies in the inherent complexity of impedance measurement and interpretation. Impedance signals include contributions from multiple system components, requiring careful analysis across frequencies and circuit configurations to deconvolve individual contributions [1]. Furthermore, optimal measurement frequencies and circuit parameters vary with the target analyte, necessitating comprehensive optimization to achieve maximum analytical performance [30]. This multi‐parameter optimization challenge has been addressed in other spectroscopic modalities using machine learning approaches; for instance, SERS‐based EV analysis combined with artificial intelligence has achieved classification accuracies exceeding 95% for distinguishing cancer‐derived EVs [24], yet the integration of AI into impedance analysis remains largely unexplored.

The second significant barrier is the high intrinsic heterogeneity of EVs. EV sizes span a broad range, making it difficult to obtain uniform measurement signals. Additionally, substantial heterogeneity in surface protein expression [31] fundamentally compromises analytical precision, regardless of the detection method employed [32]. The absence of commercially available, traceably characterized reference materials with defined internal cargo and surface protein composition [33] further complicates this challenge. The magnitude of this standardization gap has been demonstrated by recent interlaboratory comparison studies, in which EV concentration measurements varied by up to two orders of magnitude across different instruments and laboratories, even when identical samples were analyzed [34]. Such variability underscores the critical need for standardized reference materials that can enable calibration and harmonization of analytical methods across all EV analysis platforms, not only impedance‐based approaches.

To address the lack of standardized reference materials, several strategies for producing model vesicles have been explored. Synthetic vesicles assembled from defined lipid mixtures offer precise compositional control [35, 36, 37, 38, 39], but inherently lack the complex membrane protein landscape of cell‐derived EVs, limiting their utility as alternatives for surface protein analysis. Cell‐based production methods, such as serial extrusion, yield nanovesicles (NVs) whose plasma membrane‐derived lipid bilayers more closely resemble those of native EVs [40]. Proteomic comparisons have shown that extrusion‐derived NVs share approximately 71% of their membrane protein composition with naturally secreted EVs from the same parent cells [40], confirming that plasma membrane protein profiles are substantially preserved during the extrusion process. Moreover, since NVs are reconstituted from the cell plasma membrane, genetic engineering of the parent cells prior to extrusion can modulate the type and abundance of surface proteins displayed on the resulting vesicles [41, 42]. This tunability of surface protein expression suggests that engineered NVs could also serve a complementary role as model systems for EV surface protein analysis, although the development of such reference platforms with precisely defined membrane protein configurations remains unexplored.

The convergence of these challenges, including complex multidimensional impedance data requiring systematic optimization, heterogeneous biological samples lacking standardized references, and the need for real‐time analysis of dynamic membrane processes, defines the unmet needs addressed by the current study.

Here, an integrated platform is introduced that addresses these challenges by combining engineered nanovesicles displaying controlled protein oligomeric states with machine‐learning‐optimized impedance spectroscopy (Figure 1). Cell‐derived nanovesicles (NVs) were produced from HeLa cells expressing membrane‐anchored amyloid‐β42 (Aβ42) through serial extrusion, which simultaneously yields uniform vesicle sizes and preserves plasma membrane composition, generating vesicles with defined surface protein configurations and minimal size heterogeneity (Figure 1A). Aβ42 was strategically selected as a model surface protein to validate the platform's ability to quantify surface protein heterogeneity, and HeLa cells were chosen as the host cell line since they do not natively express either Aβ42 or mCherry, the two main components of the fusion protein constructs, ensuring that all detected signals originate exclusively from the engineered constructs. Furthermore, the well‐characterized surface proteome of HeLa cells [43, 44] provides a reliable reference framework for interpreting engineered membrane constructs. Beyond its clinical relevance to neurodegenerative diseases, Aβ42 provides an ideal validation system because it exhibits well‐characterized oligomerization dynamics and enables controlled variation in surface protein density and oligomeric state without altering membrane composition. Using genetic constructs encoding 1, 3, or 9 copy repeats of Aβ42, nanovesicles displaying monomer, trimer, and nonamer configurations were generated, along with control vesicles, establishing a series of reference standards with precisely defined surface protein states (Figure 1B).

**FIGURE 1:**
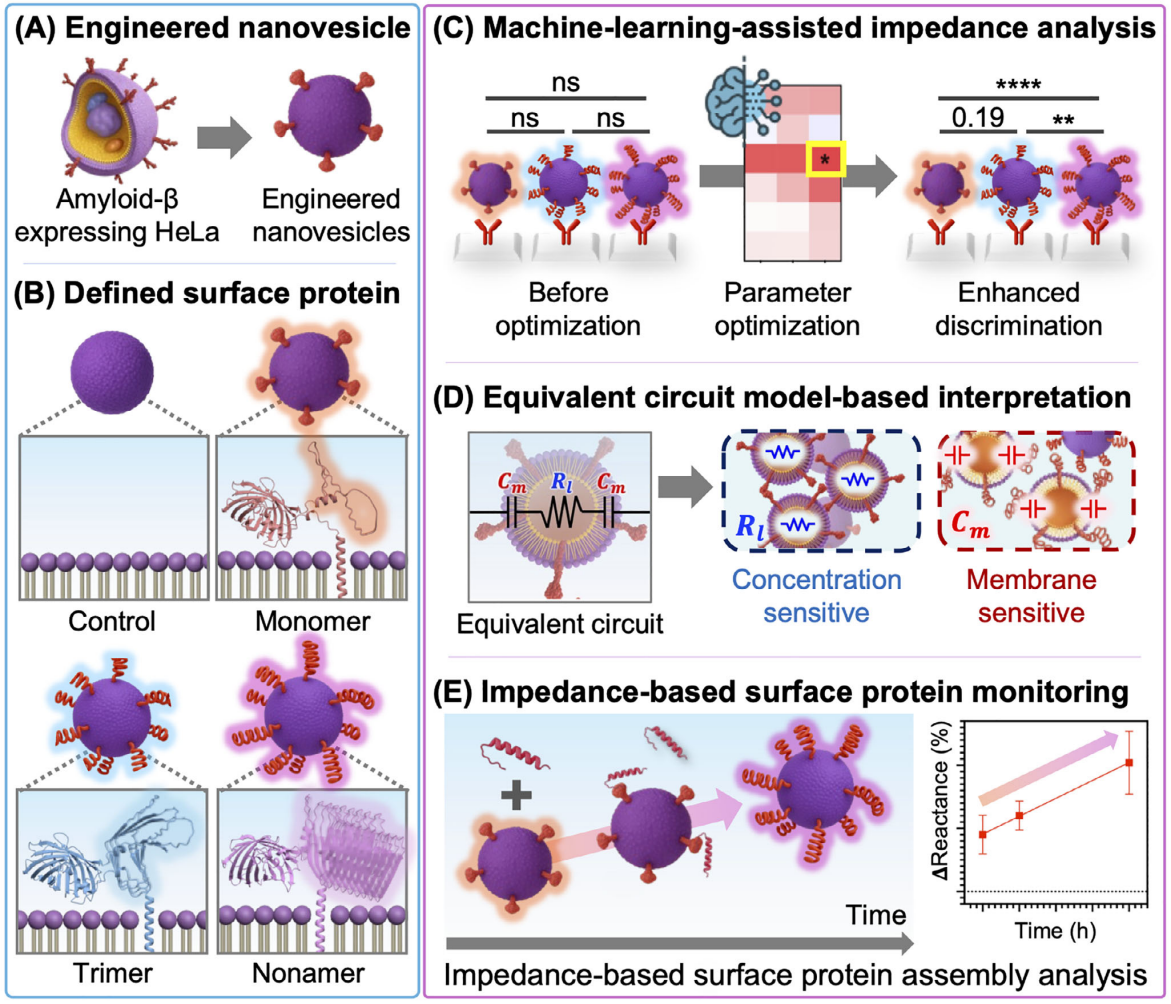
Schematic overview of the ML‐optimized impedance platform for EV surface protein analysis. (A) Generation of engineered nanovesicles from Aβ42‐expressing HeLa cells. (B) Nanovesicles with defined surface protein configurations: control, monomer, trimer, and nonamer of Aβ42. (C) ML‐assisted impedance analysis enables enhanced discrimination of oligomeric states after parameter optimization (ns, not significant; **p < 0.01; ****p < 0.0001). (D) Equivalent‐circuit interpretation: lumenal resistance (R_l_) reflects concentration sensitivity, and membrane capacitance (C_m_) reflects membrane protein composition. (E) Time‐resolved analysis of surface protein assembly dynamics using reactance changes.

Through machine‐learning (ML) guided analysis of impedance spectra across a broad frequency range, optimal measurement conditions were identified that distinguish vesicles by the oligomeric state of their surface proteins. Systematic evaluation of multiple frequency‐feature combinations using ML classifiers revealed that parameter optimization improved discrimination, with statistical significance (Figure 1C). These electrical signatures were mechanistically interpreted through equivalent‐circuit modeling, which showed that luminal resistance primarily reflects vesicle concentration, while membrane capacitance correlates with surface protein composition and oligomerization state (Figure 1D). This deconvolution of concentration‐dependent and composition‐dependent contributions provides interpretable parameters that link electrical measurements to the physical properties of vesicle membranes. The platform further enables time‐resolved, label‐free analysis to directly track protein assembly dynamics on vesicle membranes, providing insights into oligomerization (Figure 1E).

This work establishes a standardizable, universally applicable framework for vesicle surface analysis. By demonstrating that engineered nanovesicles with defined protein configurations can serve as tunable reference standards, and that equivalent‐circuit parameters derived from ML‐optimized impedance measurements correlate with membrane protein states, this approach offers a generalizable methodology for label‐free quantification of vesicle surface heterogeneity. The platform addresses critical gaps in current EV analysis: it enables detection of oligomerization states without requiring lysis or specialized conformation‐specific antibodies, provides defined reference materials for method standardization, and integrates ML to systematically optimize the interpretation of complex impedance data. These advances have broad implications for biomarker discovery, therapeutic vesicle engineering, and the development of EV analysis methods, while also providing tools for fundamental studies of membrane‐associated protein dynamics relevant to neurodegenerative disease mechanisms.

## 2 Results

### 2.1 Generation and Characterization of Aβ42‐Displaying Cell‐Derived NVs

To enable the display of different numbers of Aβ42 repeats on nanovesicles, HeLa cells were engineered to express fusion proteins containing 1, 3, or 9 copies of Aβ42 on their plasma membrane. Each fusion protein, Aβ42‐M (monomer), Aβ42‐T (trimer), and Aβ42‐N (nonamer), was constructed with a transmembrane domain (TM) from type II transmembrane protease serine 6 (TMPRSS6) to anchor the protein in the membrane, an extracellular Myc tag and mCherry for monitoring expression and detection by Western blot, and a C‐terminal glycine‐serine (GS) linker followed by the Aβ42 repeat sequence. The domain architecture is illustrated in Figure 2A.

**FIGURE 2:**
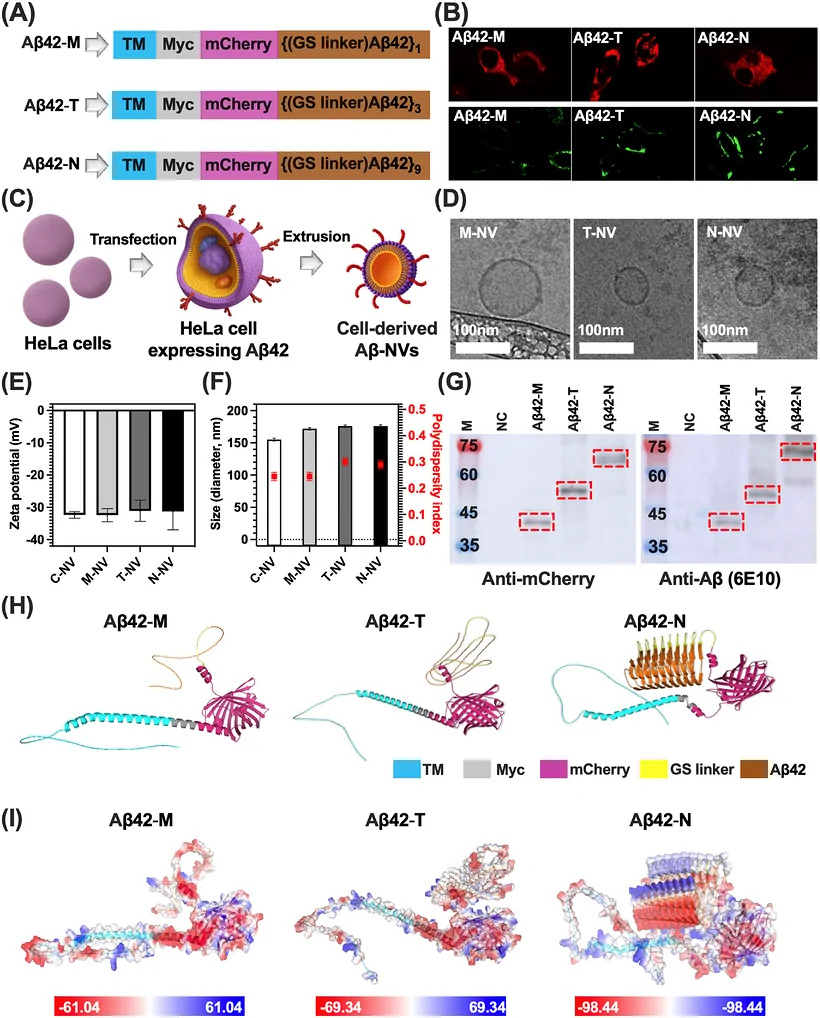
Generation and characterization of Aβ42‐NVs. (A) Schematic representation of the fusion protein domain architecture. Each construct contains a TM domain for membrane anchorage, followed by an extracellular Myc tag, mCherry fluorescent protein, a GS linker, and the specified number of Aβ42 repeats. (B) Confocal fluorescence microscopy images of HeLa cells expressing Aβ42 fusion proteins. Upper panel: mCherry signals confirming membrane localization of all three constructs. Lower panel: Immunofluorescence staining using anti‐β‐amyloid 1–42 antibody (6E10) and IgG Fc conjugated to Alexa Fluor 488, providing additional validation of Aβ42 expression at the cell surface. (C) Workflow schematic illustrating the generation of cell‐derived Aβ‐NVs from transfected HeLa cells through extrusion, producing nanovesicles with membrane‐bound Aβ42. (D) Representative cryo‐TEM images showing spherical morphology for all Aβ‐NV types. Scale bars: 100 nm. (E) Zeta potential measurements showing preserved membrane integrity across all vesicle types: C‐NV, 32.3 ± 1.0 mV; M‐NV, 32.4 ± 2.0 mV; T‐NV, 31.1 ± 3.2 mV; N‐NV, 31.3 ± 5.7 mV. Data represent mean ± SD (*n* = 4 for each group). (F) DLS analysis revealing uniform size distributions. Mean diameters: C‐NV, 155 ± 2.2 nm; M‐NV, 172 ± 2.0 nm; T‐NV, 175 ± 1.8 nm; N‐NV, 176 ± 1.9 nm. All samples exhibited a polydispersity index (PDI) < 0.3, indicating monodisperse populations. Data represent mean ± SD (*n* = 4 for each group).(G) Western blotting using anti‐mCherry and anti‐Aβ (6E10) antibodies. Aβ42‐M and Aβ42‐T were detected at expected molecular weights, while Aβ42‐N appeared at approximately 70 kDa instead of the predicted 90 kDa, suggesting structural compactness. (H) AlphaFold2‐predicted structures of Aβ42‐M, Aβ42‐T, and Aβ42‐N fusion proteins. (I) Surface charge distribution reveals increased charge heterogeneity and polarization in Aβ42‐N compared to the more uniform distributions in Aβ42‐M and Aβ42‐T. Charge scale is expressed in k_B_T e_c_^−1^.

Fusion proteins were expressed in HeLa cells under a constitutive promoter, and their expression and membrane localization were monitored using both fluorescence microscopy and immunofluorescence staining. Distinct mCherry fluorescence signals were observed along the plasma membrane for all constructs (Figure 2B, upper panel), and immunofluorescence staining with anti‐β‐amyloid antibody further confirmed correct expression and localization of each Aβ42 variant (Figure 2B, lower panel).

Next, nanovesicles displaying different repeats of Aβ42 (Aβ‐NVs) were produced according to the workflow via extrusion, as shown in Figure 2C [45]. HeLa cells expressing Aβ42‐M, Aβ42‐T, and Aβ42‐N were utilized to generate nanovesicles (denoted M‐NV, T‐NV, and N‐NV), while vesicles from non‐transfected cells (C‐NV) served as a negative control.

The characterization of the four types of nanovesicles was performed using a combination of biophysical and molecular approaches. First, the physical properties of the NVs were evaluated to confirm that all four vesicle types were generated with minimal size heterogeneity and intact membrane architecture comparable to native EVs. Dynamic Light Scattering (DLS) analysis indicated average sizes of 172 ± 2.0, 175 ± 1.8, and 176 ± 1.9 nm for M‐NV, T‐NV, and N‐NV, respectively. In comparison, C‐NVs measured 155 ± 2.2 nm. All three Aβ42‐presenting nanovesicle types exhibited a uniform size distribution and were approximately 20 nm larger than C‐NVs (Figure 2F), suggesting that increasing the number of Aβ42 repeats did not significantly alter the overall vesicle dimensions. The polydispersity index (PDI) was below 0.3, indicating a well‐dispersed and uniform particle population. The Zeta potential (ZP) measurements yielded values of 32.3 ± 1.0, 32.4 ± 2.0, 31.1 ± 3.2, and 31.3 ± 5.7 mV for C‐NV, M‐NV, T‐NV, and N‐NV, respectively, reflecting preserved membrane integrity regardless of the Aβ variant (Figure 2E).

Cryogenic transmission electron microscopy (cryo‐TEM) imaging revealed a spherical morphology and an intact membrane architecture across all nanovesicle types, consistent with the presence of all three fusion proteins (Figure 2D).

Following the analysis of physical properties, the molecular composition of the nanovesicles was examined to confirm the presence and differential expression of engineered Aβ42 fusion proteins. As the NVs were produced by extrusion rather than secreted through endosomal pathways, characterization focused on the engineered constructs rather than canonical EV markers. Western blotting was performed using two complementary antibodies, anti‐Aβ (6E10 clone), which detects the Aβ42 domain and enables verification of the target protein across the vesicle types, and anti‐mCherry, which recognizes the reporter domain common to all three fusion protein constructs and serves as a cross‐validation marker for overall construct expression. Both antibodies confirmed that the Aβ42 fusion proteins were successfully incorporated into the nanovesicles with the differential expression pattern (Figure 2G).

Aβ42‐M and Aβ42‐T were observed at their expected molecular weights, whereas Aβ42‐N was detected at approximately 70 kDa instead of the predicted 90 kDa, suggesting possible aggregation or conformational compactness during SDS‐PAGE. To further investigate this observation, the three‐dimensional structures of Aβ42‐M, Aβ42‐T, and Aβ42‐N were predicted using AlphaFold2 [46, 47], providing high‐confidence structural models for each fusion protein (Figure 2H).

Structural analysis using PyMOL revealed that increasing the number of Aβ42 repeats led to greater structural compactness, with a particularly pronounced effect observed in the Aβ42‐N construct. This increased compactness may explain the lower apparent molecular weight of Aβ42‐N observed in Western blotting, likely due to aggregation or conformational changes associated with higher oligomeric states. Furthermore, structural predictions indicated that Aβ42‐N exhibited markedly increased surface charge heterogeneity and a highly polarized electrostatic distribution compared to the more moderate and stable profiles of Aβ42‐M and Aβ42‐T (Figure 2I). Such polarization may contribute to distinct aggregation behaviors or influence electrical measurements, in line with previous reports that charge polarization enhances aggregation in higher‐order oligomers [48, 49].

Together, these results confirm the successful generation of nanovesicles expressing Aβ42‐M, Aβ42‐T, and Aβ42‐N, establishing a set of reference nanovesicles with precisely defined surface protein configurations. The key differences in surface charge properties and aggregation tendencies among the three fusion proteins, combined with the molecular, structural, and biophysical characterization presented here, provide a solid foundation for subsequent investigation of the electrical properties of these engineered nanovesicles.

### 2.2 Machine‐Learning‐Guided Impedance Analysis of Aβ‐NVs

#### 2.2.1 Quantitative Measurement of Aβ‐NVs with Interdigitated Microelectrodes

An interdigitated microelectrode (IME) biosensor integrated with a polydimethylsiloxane (PDMS) microchannel was fabricated to enable electrical analysis of the generated Aβ‐NVs (Figure 3A). The device features four interdigitated microelectrode arrays and PDMS microchannels to precisely measure the impedance of Aβ‐NVs (Figure 3B).

**FIGURE 3:**
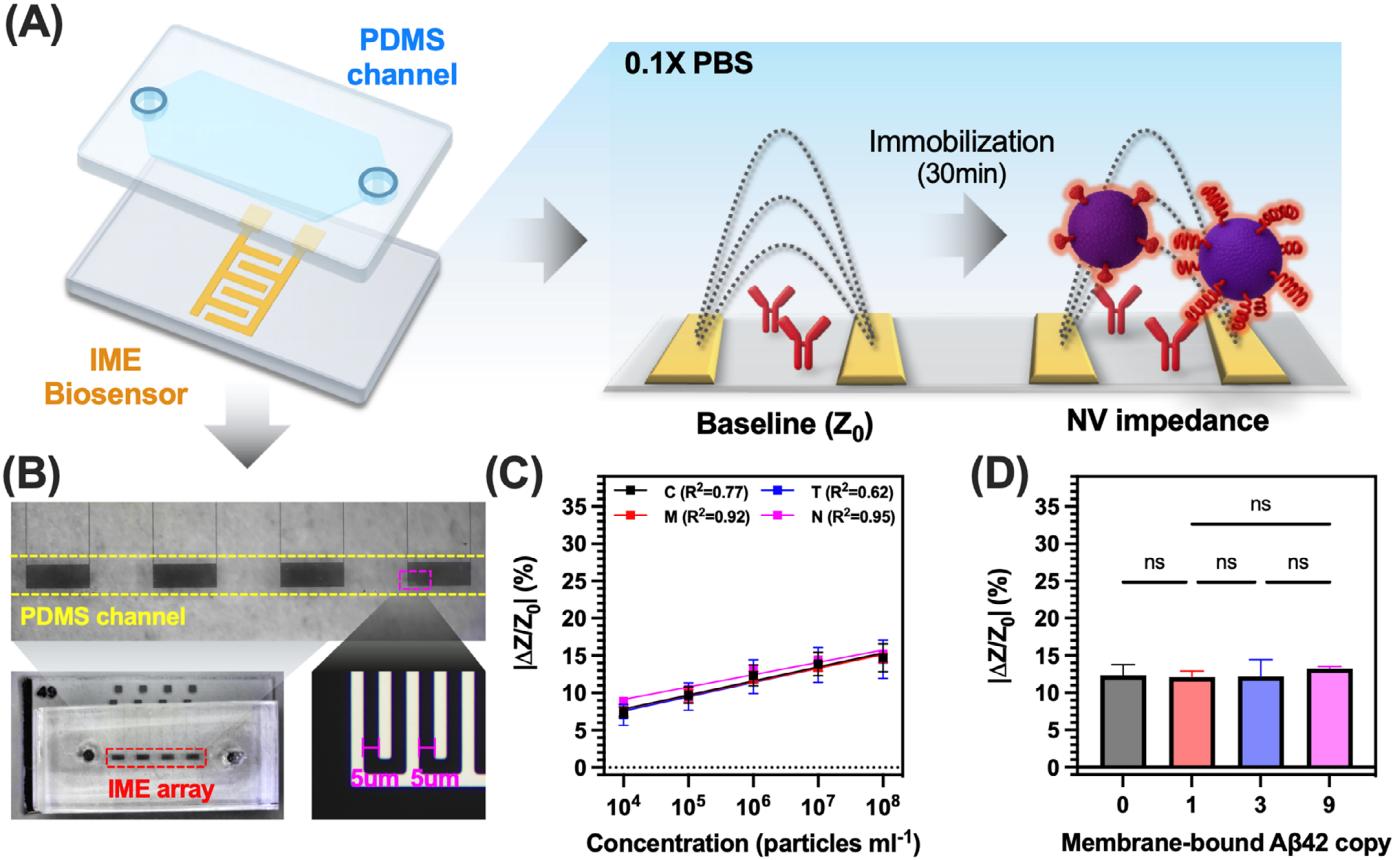
IME biosensor‐based impedance analysis of Aβ‐NVs. (A) Schematic illustration of the IME biosensor platform for Aβ‐NV characterization. Impedance measurements were performed in 0.1× PBS following antibody immobilization. Baseline impedance (Z_0_) was measured before NV capture, followed by 30‐minute incubation for vesicle immobilization and subsequent impedance measurement. (B) Photograph of the IME biosensor. Four interdigitated microelectrode arrays (5 µm gap, 5 µm width) were aligned with the central PDMS microchannel. (C) Dose‐response curves showing impedance change (|ΔZ/Z_0_|) at 100 Hz as a function of vesicle concentration for all Aβ‐NV types. Semi‐logarithmic linear responses were observed across concentrations ranging from 10^4^ to 10^8^ particles mL^−1^. Linear regression R^2^ values: C‐NV = 0.77, M‐NV = 0.92, T‐NV = 0.62, N‐NV = 0.95. Data represent mean ± SD (*n* = 12 for each concentration). (D) Comparison of |ΔZ/Z_0_| at 100 Hz among different Aβ‐NV types at 10^6^ particles mL^−1^, categorized by the number of membrane‐bound Aβ42 molecules (0, 1, 3, and 9 copies). No significant differences were observed between groups (ns, ordinary one‐way ANOVA, F(3,44) = 1.747, *p* = 0.1713; Tukey's post‐hoc test: 0 vs 1, *p* = 0.9659; 1 vs. 3, *p* = 0.9878; 3 vs. 9, *p* = 0.2470; 1 vs. 9, *p* = 0.1926). Data represent mean ± SD (*n* = 12 per group).

Impedance analysis of Aβ‐NVs was performed across a frequency range of 10 Hz to 1 MHz. Particular attention was given to the response at 100 Hz to evaluate the IME biosensor's detectability. At this frequency, the impedance change (|ΔZ/Z_0_|) displayed a semi‐logarithmic linear dose response over vesicle concentrations ranging from 1 × 10^4^ to 1 × 10^8^ particles mL^−1^ for all Aβ‐NV types, Control (C‐NVs), Monomer (M‐NVs), Trimer (T‐NVs), and Nonamer (N‐NVs), demonstrating the biosensor's quantitative sensing capabilities (Figure 3C).

Comparison of the |ΔZ/Z_0_| magnitudes among different Aβ‐NV types at a representative concentration of 1×10^6^ particles ml^−1^ (the mid‐range of tested concentrations) showed no significant difference between the groups (Figure 3D). Therefore, to improve discrimination between Aβ‐NV types based on impedance measurements, a machine‐learning‐guided approach was employed to identify the most informative combination of frequency and impedance features.

#### 2.2.2 Machine‐Learning Optimization for Impedance Analysis of Aβ‐NVs

To determine the optimal frequency‐feature pair, 18 combinations were evaluated, consisting of three impedance features (Δ|Z/Z_0_|, Δ|R/R_0_|, Δ|X/X_0_|) measured at six frequencies (ranging from 10 Hz to 1 MHz, spaced by orders of magnitude).

For each combination, three complementary performance metrics were calculated (Figure 4A): (1) the linearity score (Score_Linearity_), representing the calibration quality across concentrations, was calculated as the R^2^ value from a semi‐logarithmic regression and reflects the biosensor's detectability; (2) the order score (Score_Order_), which assesses whether the average signal values correspond to the expected order of Aβ42 peptide length on the vesicles (C‐NV, M‐NV, T‐NV, N‐NV, corresponding to 0, 1, 3, and 9 repeats), was determined using Spearman's rank correlation coefficient between the known order and the measured group means, a perfect monotonic sequence scores 1, while a completely disordered sequence approaches 0; and (3) the classification score (Score_Classification_), which quantifies the ability to distinguish among the four vesicle types, was calculated as the average of accuracy and F1‐score from six machine learning classifiers (ExtraTrees, Random Forest, Gradient Boosting, Logistic Regression, Support Vector Machine, and k‐Nearest Neighbor) trained and tested on each feature‐frequency pair. All scores were normalized to a 0–1 range and combined into a comprehensive score (Score_Comprehensive_) using a weighted mean, as described in Equation (1).

(1) $$\begin{array}{cc} Score_{Comprehensive} & =W_{1}\;\times Score_{Linearity}+W_{2}\times Score_{Order}\\ & \;+W_{3}\times Score_{Classification} \end{array}$$

**FIGURE 4:**
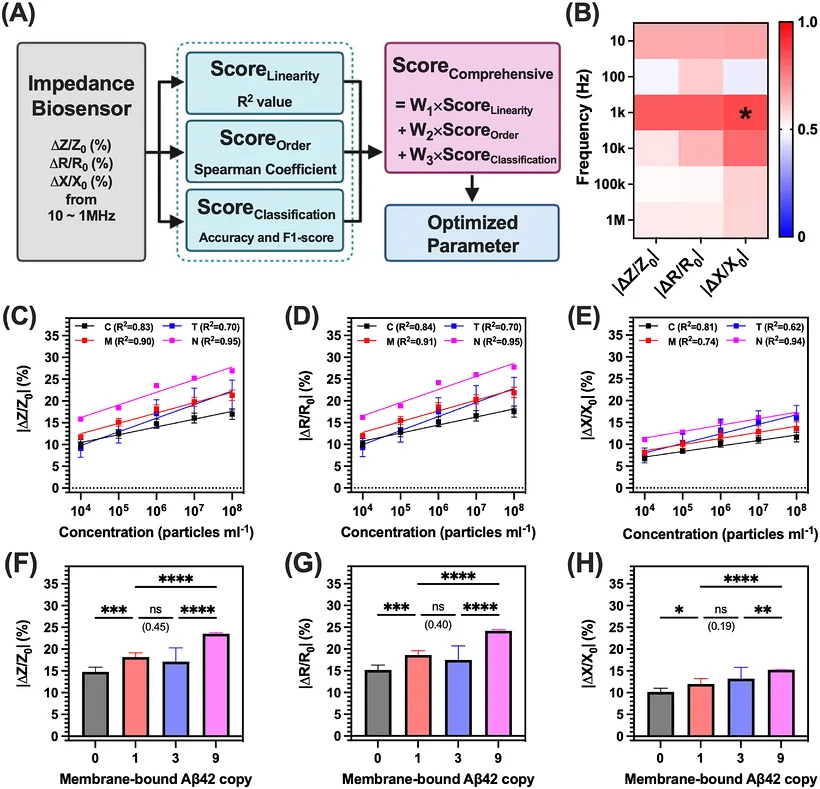
Machine‐learning–guided optimization of impedance analysis for membrane Aβ of NV. (A) Flow chart of the ML classifier approach for identifying optimal analytical conditions. Eighteen frequency‐feature combinations were comprehensively evaluated using three performance metrics: Score_Linearity_ (R^2^ value from semi‐log regression), Score_Order_ (Spearman coefficient assessing monotonic correspondence to Aβ42 copy number), and Score_Classification_ (average of accuracy and F1‐score from 6 ML classifiers). A Score_Comprehensive_ was calculated as the weighted mean (W_1_:W_2_:W_3_) of these metrics. (B) Heatmap of comprehensive scores with balanced weighting (W_1_:W_2_:W_3_ = 1:1:1). The reactance change (Δ|X/X_0_|) at 1 kHz achieved the highest score (asterisk), identifying this as the optimal condition for Aβ‐NV analysis. (C‐E) Dose‐response curves at 1 kHz for (C) Δ|Z/Z_0_|, (D) Δ|R/R_0_|, and (E) Δ|X/X_0_|. All features exhibited semi‐logarithmic linear responses across 10^4^–10^8^ particles mL^−1^. Linear regression R^2^ values for C‐NV, M‐NV, T‐NV, and N‐NV were: (C) 0.83, 0.90, 0.70, 0.95; (D) 0.84, 0.91, 0.70, 0.95; (E) 0.81, 0.74, 0.60, 0.94, respectively. Data represent mean ± SD (*n* = 12 for each concentration). (F‐H) Comparison of impedance features at 10^6^ particles mL^−1^ categorized by membrane‐bound Aβ42 copy number. (F) Δ|Z/Z_0_| showed significant differences (****p < 0.0001, ordinary one‐way ANOVA, F(3,44) = 53; Tukey's: 0 vs 1, *p* = 0.0001; 1 vs. 3, *p* = 0.4547; 3 vs. 9, *p* < 0.0001; 1 vs. 9, *p* < 0.0001). (G) Δ|R/R_0_| displayed similar trends (****p < 0.0001, F(3,44) = 55; Tukey's: 0 vs. 1, *p* = 0.0001; 1 vs. 3, *p* = 0.4064; 3 vs 9, *p* < 0.0001; 1 vs. 9, *p* < 0.0001). (H) Δ|X/X_0_| exhibited monotonic increases with Aβ42 copy number (****p < 0.0001, F(3,44) = 24; Tukey's: 0 vs. 1, *p = 0.0256; 1 vs. 3, *p* = 0.1858; 3 vs. 9, **p = 0.0095; 1 vs. 9, ****p < 0.0001). Data represent mean ± SD (*n* = 12 per group).

Among all 18 combinations, the Score_Comprehensive_ with equal weights (W_1_:W_2_:W_3_ = 1:1:1) was highest for the reactance change (Δ|X/X_0_|) measured at 1 kHz, identifying this as the optimal analytical condition (Figure 4B, asterisk). This optimal point remained consistent across different weighting schemes that prioritized linearity (Figure S1A), order (Figure S1B), or classification (Figure S1C), indicating that 1 kHz is robustly optimal regardless of the specific analytical objective.

To validate the selected frequency‐feature pair, concentration‐dependent responses at 1 kHz were measured for Δ|Z/Z_0_|, Δ|X/X_0_|, and Δ|R/R_0_|. All three features exhibited clear log‐linear trends across vesicle concentrations from 1×10^4^ to 1×10^8^ particles mL^−1^ for each vesicle type (Figure 4C–E), confirming effective quantification at 1 kHz. However, their sensitivity to the number of Aβ42 peptides differed: Δ|X/X_0_| displayed a strictly monotonic increase corresponding to the sequence C < M < T < N (Figure 4H), whereas Δ|Z/Z_0_| and Δ|R/R_0_| only partially reflected this order and did not consistently distinguish all groups (Figure 4F,G). Thus, while all features were responsive to concentration, reactance (X) provided the most accurate discrimination of vesicles based on the number of membrane‐bound Aβ42 peptides.

### 2.3 Equivalent‐Circuit Modeling for Aβ‐NVs

Impedance changes, especially the sensitivity of reactance to Aβ42, were analyzed using the modeled equivalent circuit. In 0.1× PBS, the IMEs are characterized by the solution path Z_s_ (R_s_ || C_s_) and the electrode double‐layer capacitances C_dl_, providing the baseline impedance of the IME biosensor Z_A_ (Figure 5A).

(2) $$Z_{A}=Z_{s}+\frac{2}{j\omega C_{dl}}$$

**FIGURE 5:**
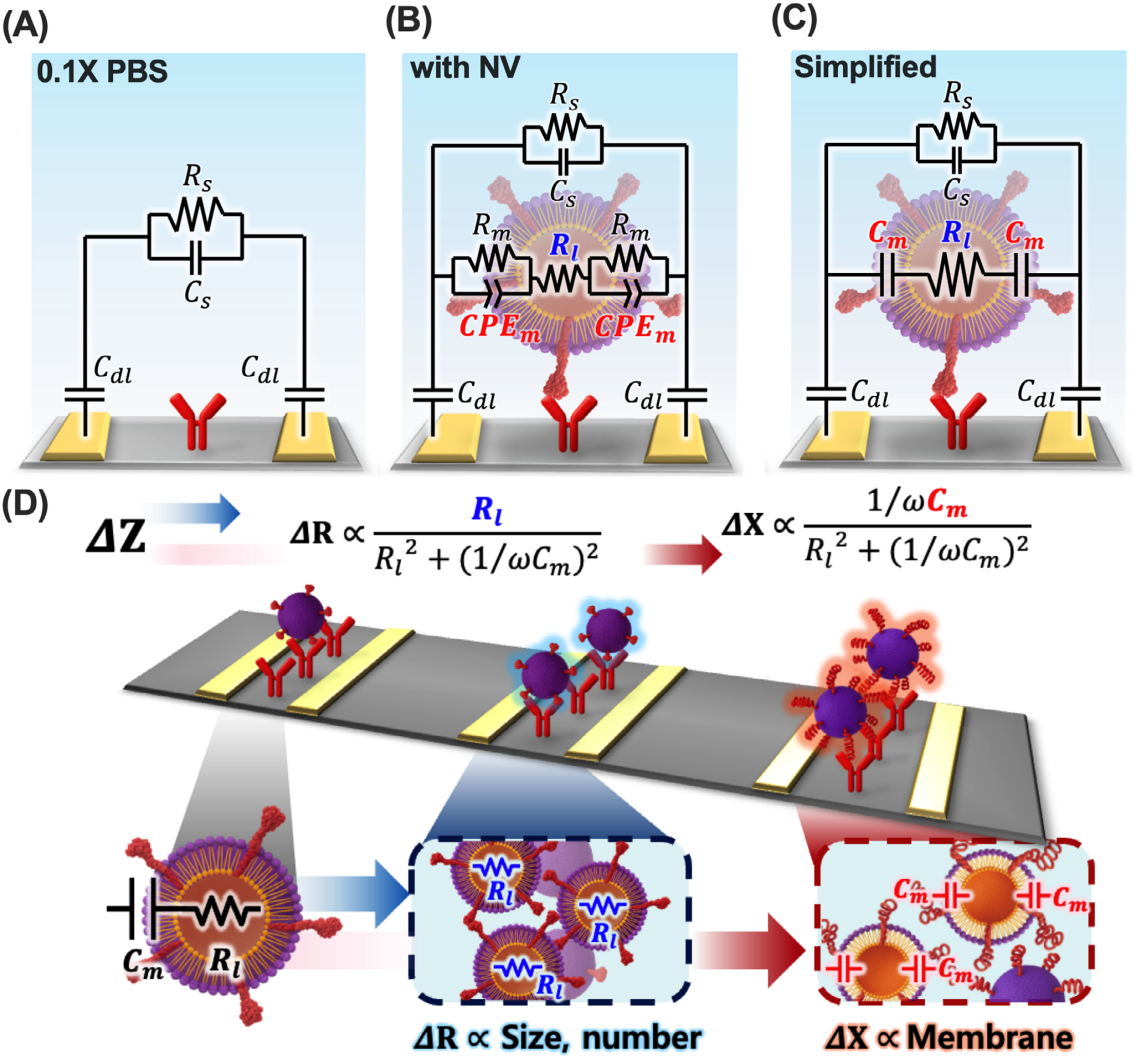
Equivalent‐circuit model for interpretable Aβ‐NV impedance analysis. (A) Baseline equivalent circuit model of the IME biosensor in 0.1× PBS before vesicle capture. The circuit comprises the electrode double‐layer capacitances (C_dl_) at each electrode surface and the electrolyte solution path Z_s_, represented as the parallel combination of solution resistance (R_s_) and solution capacitance (C_s_). (B) Equivalent circuit model after Aβ‐NV immobilization. The vesicle branch is introduced in parallel with the solution path, consisting of the luminal resistance (R_l_) in series with the membrane impedance Z_m_. The membrane pathway is modeled as a constant phase element (CPE_m_) in parallel with a leakage resistance (R_m_), representing the semipermeability of the plasma membrane. (C) Simplified equivalent circuit model obtained through first‐order approximation for intuitive parameter interpretation. The membrane element CPE_m_ is idealized as a pure capacitor (C_m_). (D) Schematic illustration of impedance change decomposition into resistance (ΔR) and reactance (ΔX) components. ΔR is predominantly influenced by R_l_, which scales with vesicle concentration (parallel pathway increase) or lumen volume (individual R_l_ increase). ΔX is primarily governed by C_m_, reflecting changes in membrane dielectric properties and protein composition.

Upon immobilization of Aβ‐NVs between the electrodes, an additional vesicle branch is placed in parallel with the solution path (Figure 5B). The equivalent circuit model of the vesicle‐immobilized surface was designed to refer to the equivalent circuit models for single cell impedance analysis [9, 50, 51], since the cytoplasm and plasma membranes were produced from its originating cell. The vesicle branch comprises the luminal resistance R_l_ in series with the membrane pathway, modeled as *Z_m_* =  (*CPE_m_* ||*R_m_*), and the corresponding admittance is *Y_ves_* = 1/(*R_l_* + *Z_m_*) . The total impedance after NVs binding is

(3) $$Z_{B}={\left(Z_{A}^{-1}+Y_{ves}\right)}^{-1}$$

so the complex change is given precisely by Equation (4).

(4) $$\Delta Z=Z_{B}\;-\;Z_{A}=-\frac{Z_{A}^{2}Y_{ves}}{1+Z_{A}Y_{ves}}$$

From the derived Equation (4), a dimensionless measure of how strongly the vesicle branch perturbs the baseline is obtained by defining the coupling magnitude μ in Equation (5)

(5) $$\mu \equiv |Z_{A}Y_{ves}|$$

Physically, μ compares the admittance of the vesicle pathway to that of the baseline sensor. If μ ≪ 1, this indicates the vesicle branch is a small perturbation. Hence, when μ is well below unity, the first‐order approximation can be used to significantly simplify interpretation without altering the qualitative dependence on vesicle parameters, as shown in Equation (6).

(6) $$\Delta Z\approx -Z_{A}^{2}Y_{ves}$$

For direct parameter intuition, the membrane element CPE_m_ was further idealized as a capacitor (Figure 5C), giving

(7) $$Z_{ves}=R_{l}+\frac{1}{j\omega C_{m}}$$

(8) $$\begin{array}{c} Y_{ves}=\frac{1}{R_{l}+\left(1/j\omega C_{m}\right)}=\frac{R_{l}}{R_{l}^{2}+{\left(1/\omega C_{m}\right)}^{2}}+j\frac{1/\omega C_{m}}{R_{l}^{2}+{\left(1/\omega C_{m}\right)}^{2}} \end{array}$$

Substitution into Equation (4) and separation into real/imaginary parts of impedance show that, after removal of baseline‐dependent scale factors, as shown in Figure 5D.

(9) $$\Delta R\propto \frac{R_{l}}{R_{l}^{2}+{\left(1/\omega C_{m}\right)}^{2}}$$

(10) $$\Delta X\propto \frac{1/\omega C_{m}}{R_{l}^{2}+{\left(1/\omega C_{m}\right)}^{2}}$$

Accordingly, the resistance change Δ*R* is expected to be mainly due to the luminal resistance R_l_, which increases with vesicle concentration or lumen size. In contrast, the reactance change Δ*X* is expected to be primarily influenced by the membrane capacitance C_m_, indicating dielectric properties and protein content. A complete, step‐by‐step derivation was provided in the Supporting Information S2.

### 2.4 Equivalent Circuit Model Fit‐Based Feature‐Sensitivities for Aβ‐NVs

To interpret the variations in impedance measured from the IME biosensor, especially the frequency‐feature pairs optimized through machine learning, the experimentally obtained impedance spectra from all four NV types across multiple concentrations were fitted using the proposed equivalent circuit model, enabling the extraction of R_l_ and C_m_. The baseline impedance (Z_A_) was used to initially estimate the parameters C_dl_ and Z_s_. Since vesicles were specifically immobilized on the glass substrate between the electrodes via antibody binding, it was assumed that C_dl_ and Z_s_ remained nearly constant following vesicle attachment (Z_B_). Subsequently, the parameters Z_m_, CPE_m_, and R_l_ of the vesicle‐branch were extracted by fitting the impedance at Z_B_, with C_dl_ and Z_s_ held fixed at the values derived from Z_A_.

The frequency dependence of the coupling magnitude (µ) revealed that µ ≪ 1 within the 100 Hz–10 kHz range (Figure S2A), indicating that the vesicle‐branch impedance could be simplified as shown in Figure 5D. In this simplified model, R_l_ primarily reflects sensitivity to vesicle concentration, while C_m_ primarily reflects sensitivity to membrane capacitance. The fitted CPE_m_ values were converted to the effective capacitance (C_eff_) at 1 kHz, enabling a direct comparison between R_l_ and C_eff_ to assess their relative sensitivity to vesicle concentration and membrane composition.

Analysis of R_l_ as a function of concentration (Figure 6A) revealed log‐linear behavior for all four vesicle types, and C_eff_ exhibited a similar log‐linear dependence (Figure 6B). In both cases, immobilization of vesicles between the IME electrodes increased the number of parallel conduction paths, resulting in a decrease in R_l_ and an increase in C_eff_. These observations are consistent with the geometric arrangement of vesicles on the biosensor surface. A comparison of the semi‐logarithmic slopes (|Slope|) demonstrated that R_l_ exhibited a larger slope than C_eff_ (Figure 6C), indicating that R_l_ responds more sensitively to changes in vesicle concentration.

**FIGURE 6:**
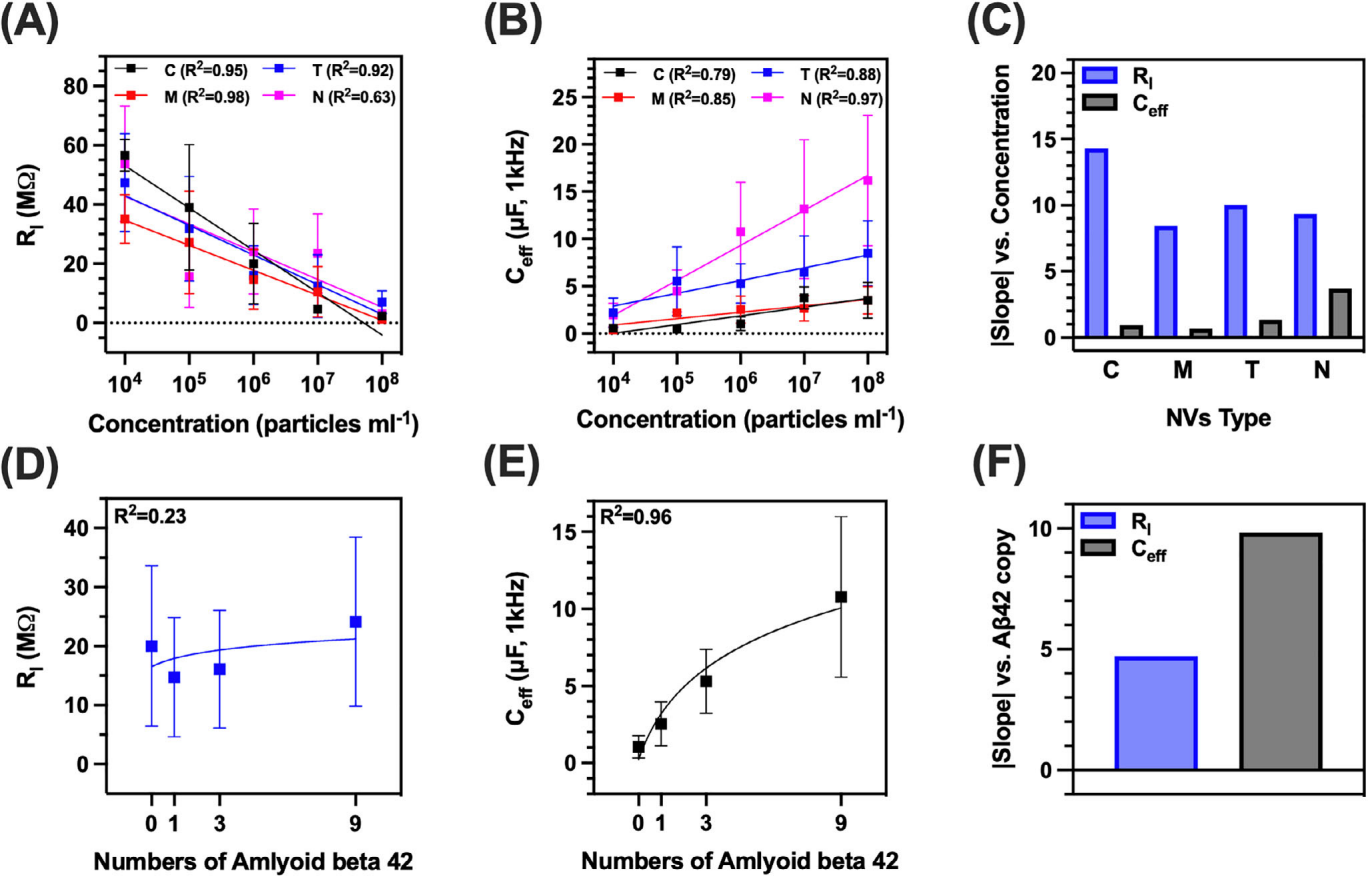
Equivalent circuit model fitting‐based sensitivities. (A) Concentration‐dependent changes in R_l_ derived from equivalent circuit fitting. All four Aβ‐NV types exhibited semi‐logarithmic linear decreases in R_l_ with increasing concentration. Linear regression R^2^ values: C‐NV, 0.95; M‐NV, 0.98; T‐NV, 0.92; N‐NV, 0.63. Data represent mean ± SD (*n* = 12 for each concentration). (B) Concentration‐dependent changes in C_eff_ at 1 kHz, calculated from fitted CPE_m_ values. All vesicle types displayed semi‐logarithmic linear increases in C_eff_ with concentration. Linear regression R^2^ values: C‐NV, 0.79; M‐NV, 0.85; T‐NV, 0.88; N‐NV, 0.97. Data represent mean ± SD (*n* = 12 for each concentration). (C) Comparison of absolute slope values from concentration‐response curves. R_l_ exhibited larger slopes than C_eff_ across all vesicle types, indicating greater sensitivity to concentration changes. (D) R_l_ as a function of membrane‐bound Aβ42 copy number at 10^6^ particles ml^−1^ and 1 kHz. No significant correlation was observed between R_l_ and Aβ42 copy (linear regression R^2^ = 0.23). Data represent mean ± SD (*n* = 12 per group). (E) C_eff_ as a function of membrane‐bound Aβ42 copy number at 10^6^ particles ml^−1^ and 1 kHz. C_eff_ increased monotonically with Aβ42 content, exhibiting strong linear correlation (R^2^ = 0.96). Data represent mean ± SD (*n* = 12 per group). (F) Comparison of absolute slope values from Aβ42‐response curves. C_eff_ displayed a significantly steeper slope than R_l_, establishing that C_eff_ is the primary reporter of membrane composition changes.

The sensitivity to membrane composition, particularly to the number of membrane proteins, was also examined. Across vesicle types (C‐NV → M‐NV → T‐NV → N‐NV; 0 → 1 → 3 → 9 membrane proteins), R_l_ showed no significant variation (Figure 6D), whereas C_eff_ increased markedly with the number of membrane proteins (Figure 6E). Comparison of the semi‐log slopes confirmed that C_eff_ was more responsive than R_l_ to changes in membrane composition (Figure 6F), consistent with the proposed model predicting that R_l_ is sensitive primarily to the lumenal path, while C_eff_ reflects the dielectric contribution of the membrane.

These findings demonstrate that the overall impedance of a vesicle encapsulates both concentration‐dependent and membrane‐dependent information. Deconvolving these components via equivalent‐circuit fitting enables selective interpretation based on the target parameter. Furthermore, since both R_l_ and C_eff_ reflect concentration, size, and membrane information, the optimal analysis frequency at which both parameters contribute maximally was identified around 1 kHz (Supporting Information S2; Figure S2B). This value corresponded well with the optimal frequency predicted by the machine‐learning–guided evaluation.

Collectively, these results support that sensitivity‐optimized parameter extraction through equivalent‐circuit modeling, combined with impedance–feature–based machine learning, provides a useful approach for impedance analysis of vesicle characteristics.

### 2.5 In Silico Prediction of Membrane‐Expressed Aβ‐Induced Capacitance Change

To clarify why the reactance measured from the IME biosensor and the capacitance (C_m_, obtained as C_eff_ from the equivalent circuit) vary depending on the number of membrane‐bound Aβ42 chains, the theoretical membrane capacitance was estimated through a structure‐based approach based on the predicted extracellular structures of the mCherry–Aβ42 constructs. The Aβ‐NVs were designed so that proteins were expressed on the outer surface of the plasma membrane (Figure 7A). The membrane‐anchored proteins' capacitance was approximated using a parallel‐plate capacitor model, where the effective area of the extracellular domain was used to define the plate area.

**FIGURE 7:**
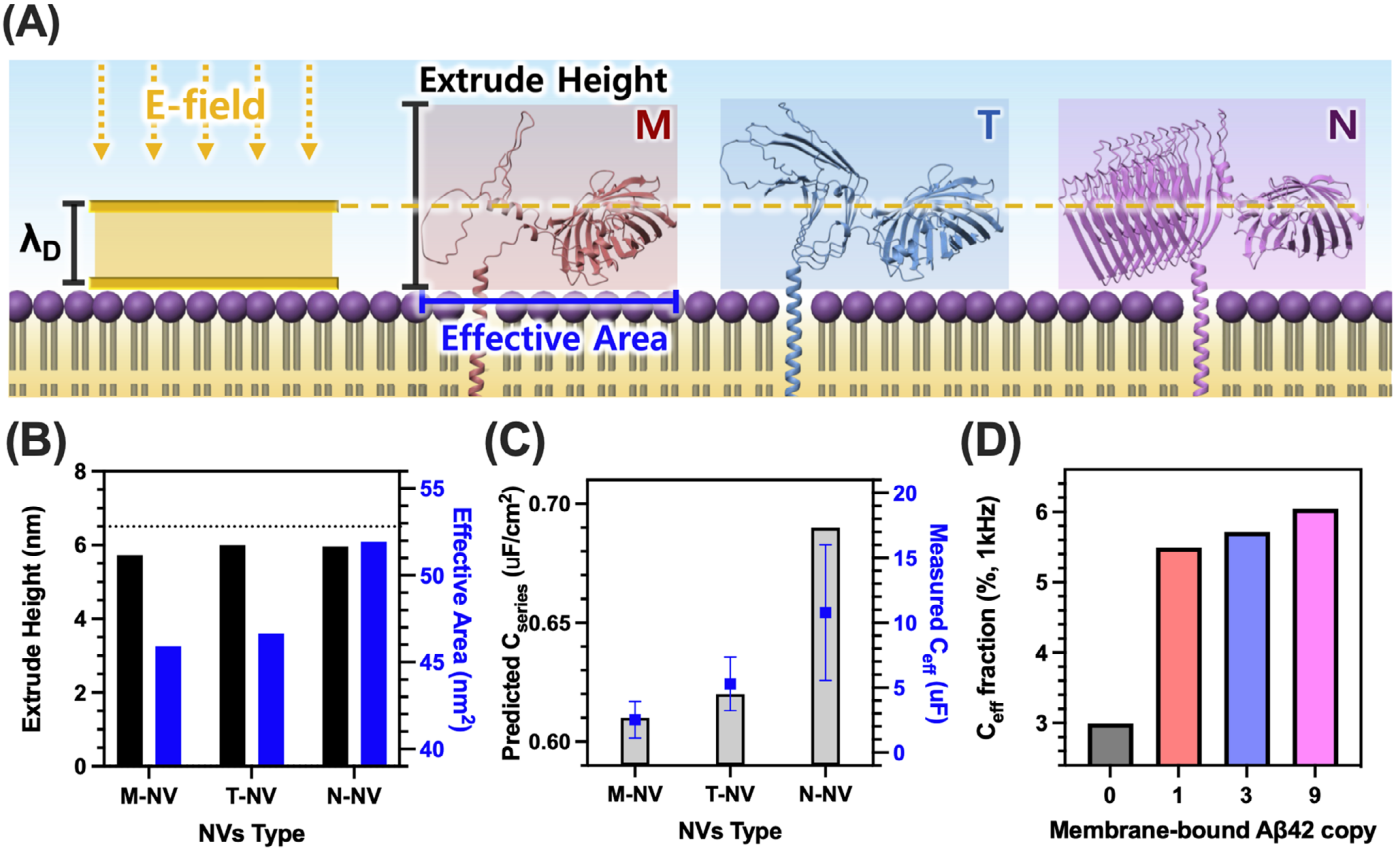
Structure‐based prediction of membrane capacitance. (A) Schematic illustration of the parallel‐plate capacitor model used to predict C_m_ based on membrane‐bound Aβ42 structures. The E‐field between IME electrodes penetrates the vesicle membrane and extracellular protein domains. (B) Effective membrane areas calculated from AlphaFold2‐predicted structures projected onto the membrane surface. The dashed black line indicates the upper limit of protein protrusion height estimated from DLS measurements. As Aβ42 copy number increases (M → T → N), the effective area occupying the membrane‐proximal region progressively expands. (C) Predicted series capacitance (C_Series_) based on effective areas compared with experimentally derived C_eff_ from equivalent circuit fitting. Data represent mean ± SD (*n* = 12 per group for experimental C_eff_ values). (D) Fractional contribution of C_eff_ to total measured impedance (Z_B_) at 1 kHz. Despite C_eff_ being the primary reporter of membrane composition, its contribution to total impedance magnitude remains below 6% across all vesicle types, with the fraction systematically increasing with membrane‐bound Aβ42 content. Data represent mean ± SD (*n* = 12 per group).

To determine the valid surface area of each construct, the amino acid sequence from mCherry to Aβ42, excluding the TM–Myc anchor that spans the plasma membrane, was modeled in AlphaFold2 for each oligomeric type (Figure S3A). Multiple docking poses of the predicted structures on the membrane surface were evaluated (Figure S3B), and the pose showing an appropriate protrusion height, consistent with the hydrodynamic diameters of C‐NV and Aβ‐NVs measured by DLS, was chosen (Figure S3C). The effective surface area, representing the “shadow” of the extracellular protein projected onto the membrane by the vertically oriented electric field, was calculated in PyMOL (Figure 7B).

Next, the relative permittivity ε_*r*_ of the proteins was estimated using the Clausius–Mossotti relation [52, 53, 54] (Equation (11)), based on their elemental composition, known atomic polarizabilities, and molecular masses of each sequence segment (mCherry–Aβ42).

(11) $$\frac{4\pi \alpha }{3V}=\frac{\varepsilon_{r}-1}{\varepsilon_{r}+1}\;$$

The static polarizability (α) of each element was used, and the molecular volume *V* was calculated from the total protein mass, with a standard protein density of 1.35 g cm^−3^ [55]. The resulting relative permittivities were estimated as 2.82 for M‐NV and T‐NV, and 2.81 for N‐NV, indicating minimal variation among the constructs.

Each protein domain was then modeled as a vertical dielectric layer connected in series to the plasma membrane in a parallel‐plate setup. The capacitance was calculated using the relevant formula, with the Debye length in 0.1× PBS (∼2.3 nm) serving as the effective dielectric thickness [56, 57]. The intrinsic protein capacitance C_protein_ (Figure S3D) was combined in series with the membrane capacitance (1.9 µF cm^−2^ for HeLa cell [58]) to determine the predicted areal capacitance C_Series_. The predicted C_Series_ increased gradually from M‐NV to N‐NV, aligning with the experimentally derived C_eff_ obtained from the equivalent‐circuit analysis (Figure 7C). This agreement between in silico structural prediction and the experimentally fitted values serves as cross‐validation, confirming that the rise in membrane capacitance corresponds to the quantitative increase in membrane‐expressing Aβ42.

However, both the predicted and experimentally derived capacitances were substantially smaller than the measured magnitude of the total impedance.

(12) $$Z_{C_{eff}}=\frac{1}{\omega C_{eff}}\;$$

(13) $$C_{eff}\;fraction=\frac{\left|Z_{C_{eff}}\right|}{\left|Z_{B}\right|}$$

When the fractional contribution of C_eff_ to the total impedance (Z_B_) was calculated using Equations (12) and (13), it accounted for less than 6% at 1 kHz across all samples, with the fraction increasing as the number of membrane proteins grew (Figure 7D). This trend suggests that membranes with higher protein density cause more significant changes in reactance. Despite the small absolute contribution, the relative changes in C_eff_ are highly sensitive to membrane protein content, making it a reliable reporter for compositional differences. The low fractional contribution of C_eff_ was consistently observed throughout the entire frequency range (Figure S4).

Overall, these results suggest that increasing effective membrane area and protein expression on the vesicle surface is associated with enhanced membrane capacitance. The structure‐based prediction and equivalent‐circuit estimation clearly explain the experimentally observed relationship between C_eff_ and membrane composition. Therefore, reactance analysis in impedance spectroscopy for vesicles is supported as a method for distinguishing vesicles with different levels of membrane‐expressed Aβ proteins by combining empirical data, circuit modeling, and structural prediction.

### 2.6 Time‐Resolved Monitoring of Aβ Oligomerization on NVs Membrane

To determine whether the reactance‐based analysis of Aβ‐expressing vesicle membranes using the IME biosensor could also be applied beyond gene‐defined Aβ expression to spontaneously grown Aβ oligomers, Aβ‐NVs were incubated with soluble Aβ to induce surface oligomerization (Figure 8A).

**FIGURE 8:**
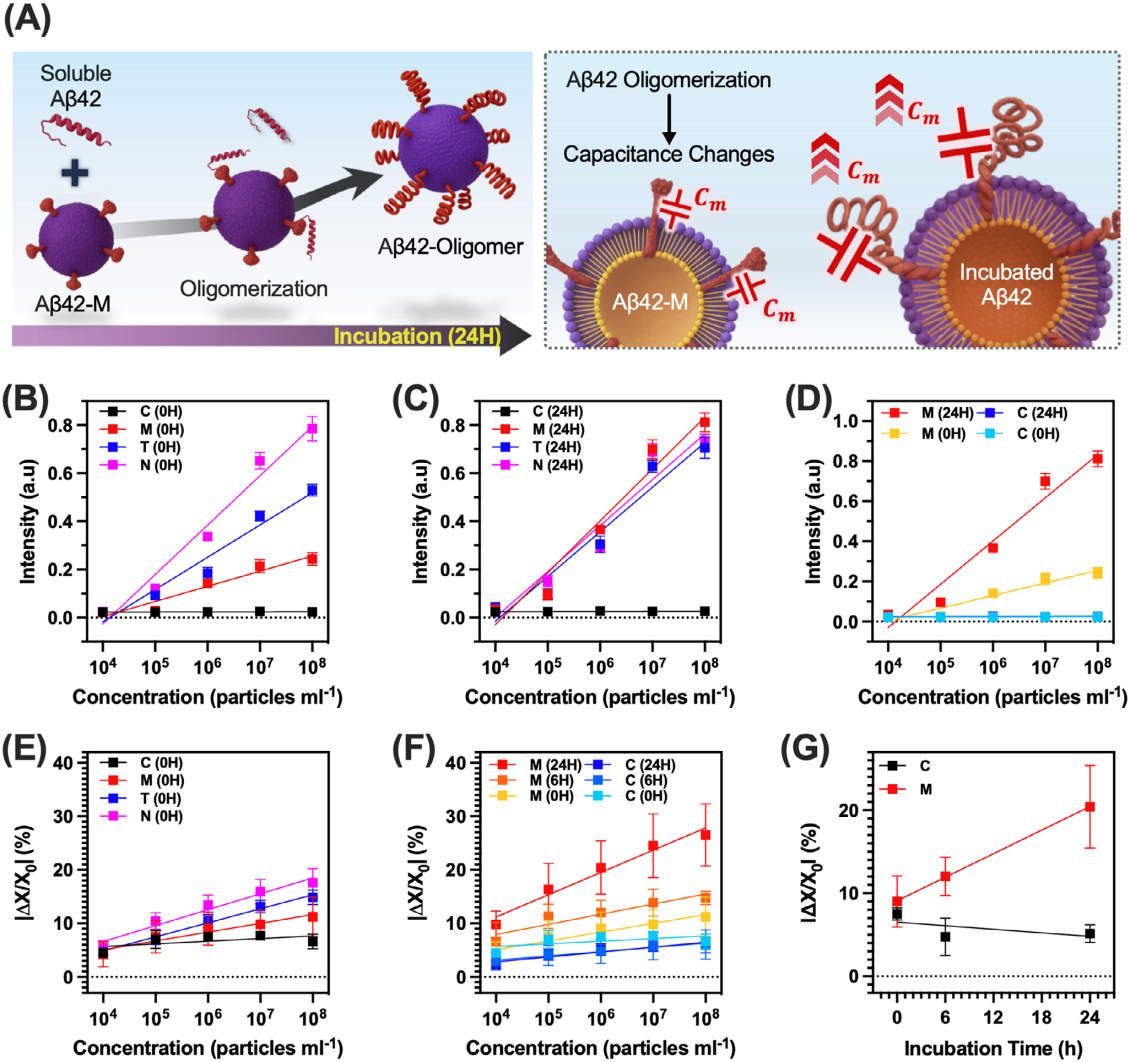
Time‐resolved oligomerization of soluble Aβ on Aβ‐NVs through impedance analysis. (A) Schematic representation of the experimental strategy for generating physiologically formed Aβ oligomers on M‐NVs. Membrane‐anchored monomeric Aβ42 served as nucleation sites for soluble Aβ42 oligomerization during incubation at 4°C. Progressive oligomer growth on the vesicle surface increases C_m_, enabling capacitance‐based monitoring of aggregation. (B) sELISA using 6E10 antibody for vesicles without soluble Aβ incubation (0 h). C‐NVs showed no signal, while all Aβ‐NV types exhibited semi‐logarithmic linear responses with vesicle concentration. Linear regression R^2^ values: C‐NV, 0.53; M‐NV, 0.94; T‐NV, 0.96; N‐NV, 0.97. Data represent mean ± SD (*n* = 3 for each concentration). (C) sELISA results after 24 h incubation with soluble Aβ42. M‐NV and T‐NV signals increased to levels comparable with N‐NVs, indicating successful oligomer formation. Linear regression R^2^ values: C‐NV, 0.32; M‐NV, 0.96; T‐NV, 0.95; N‐NV, 0.93. Data represent mean ± SD (*n* = 3 for each concentration). (D) Comparison of C‐NV and M‐NV before (0 h) and after (24 h) incubation. M‐NVs exhibited significant signal enhancement following incubation, whereas C‐NVs remained undetectable, confirming selective oligomerization on pre‐existing membrane‐bound Aβ42. Data replotted from panels B and C. (E) Δ|X/X_0_| at 1 kHz measured by IME biosensor without soluble Aβ incubation (0 h). Semi‐logarithmic linear responses were observed with increasing magnitude correlated to Aβ42 copy number. Linear regression R^2^ values: C‐NV, 0.20; M‐NV, 0.38; T‐NV, 0.90; N‐NV, 0.80. Data represent mean ± SD (*n* = 12 for each concentration). (F) Concentration‐dependent reactance changes for C‐NVs and M‐NVs following 0, 6, and 24 h incubation with soluble Aβ42. C‐NVs showed no significant changes across concentrations (R^2^ = 0.20, 0.21, 0.55 for 0, 6, 24 h), while M‐NVs exhibited progressive increases in reactance with both concentration and incubation duration (R^2^ = 0.38, 0.52, 0.59 for 0, 6, 24 h). Data represent mean ± SD (*n* = 12 for each concentration). (G) Linear relationship between incubation time and reactance change at 10^6^ particles ml^−1^. M‐NVs displayed strong linear correlation (R^2^ = 0.99) between oligomerization time and reactance change, whereas C‐NVs showed no correlation (R^2^ = 0.36). Data represent mean ± SD (*n* = 12 per time point).

To determine suitable conditions for Aβ growth without vesicle–vesicle hybridization, C‐NVs, M‐NVs, and N‐NVs were incubated for up to 168 h at 4°C or 37°C with gentle shaking. At 4°C, all vesicles remained monodisperse and morphologically intact throughout the entire monitoring period (Figure S5A), while at 37°C, progressive vesicle aggregation and size increase were observed after 24 h through DLS and cryo‐TEM (Figure S5B,C). Importantly, 4°C incubation is a well‐established protocol for selectively preparing oligomeric Aβ42 species, as distinct from fibrillar aggregates that predominate at 37°C [59, 60]. This temperature was therefore selected for subsequent incubations to simultaneously preserve vesicle integrity and promote controlled surface oligomerization.

Aβ‐NVs were then co‐incubated with 1 µM of soluble Aβ42 at 4°C for up to 24 h, followed by ultracentrifugation (UC) to remove unbound Aβ and isolate the vesicles. This concentration substantially exceeds the critical aggregation concentration (∼90 nM) reported for Aβ42 oligomer formation [61, 62], ensuring the conditions for oligomerization were satisfied. To control for potential measurement artifacts caused by incomplete removal of free Aβ or UC‐induced vesicle deformation, Aβ‐NVs that underwent immediate UC separation without incubation served as the 0 h control group. After incubation and purification, the recovered vesicles were quantified by NTA and evaluated for membrane‐associated Aβ oligomerization using sandwich ELISA (sELISA).

In sELISA assays using the 6E10 antibody, signal intensity increased with Aβ concentration in the 0 h samples (Figure 8B). After 24 h of incubation, C‐NVs remained unchanged, while M‐NVs, T‐NVs, and N‐NVs exhibited comparable signal intensities, confirming efficient oligomer formation on the vesicle surface (Figure 8C). Notably, C‐NVs showed no change before and after incubation, whereas M‐NVs displayed a clear increase in intensity, indicating that soluble Aβ selectively oligomerized on pre‐existing Aβ chains anchored to the vesicle surface (Figure 8D).

To confirm that the accumulated Aβ adopted an oligomeric conformation, an additional sELISA was performed using the anti‐oligomer antibody (A11), which selectively recognizes oligomeric Aβ species but not monomers. C‐NVs and M‐NVs were not detected in 0 h samples (Figure S6A), but after 24 h incubation, M‐NVs transitioned to A11‐positive and exhibited signal intensities comparable to those of T‐NVs and N‐NVs (Figure S6B), confirming that oligomers had formed on the M‐NV membrane surface through physiological aggregation (Figure S6C).

To determine whether these structural changes could be detected electrically, the IME biosensor was used to measure reactance variations at 1 kHz, the optimal frequency identified through ML‐guided evaluation and equivalent‐circuit analysis. Control experiments with 0 h samples confirmed stable measurement performance and reliable detection across different vesicle concentrations and Aβ levels (Figure 8E). Next, C‐NVs and M‐NVs incubated with soluble Aβ for 0, 6, and 24 h were measured (Figure 8F). While C‐NVs exhibited negligible change with incubation time, M‐NVs exhibited a progressive increase in reactance variation. When the reactance changes were plotted against incubation time at equal concentrations, the results indicated that oligomer growth on M‐NV membranes led to a measurable increase in reactance proportional to the amount of membrane‐bound Aβ (Figure 8G).

Together, optical verification using sELISA and electrical detection via reactance analysis using IME biosensor, provided consistent results that soluble Aβ42 oligomerized on membrane‐anchored Aβ42 monomers at 4°C. These findings indicate that the reactance‐based analysis on the IME biosensor can detect variations in membrane protein states, both in gene‐regulated NVs expressing predefined numbers of Aβ copies and in incubation‐induced oligomeric Aβ assemblies on the vesicle membrane, validating the analytical framework established through ML optimization and equivalent‐circuit modeling using the engineered NV standards.

## 3 Discussion and Conclusion

In this study, a universally applicable analytical framework was established for characterizing vesicle surface protein heterogeneity by integrating engineered NVs with ML‐guided impedance spectroscopy. Using Aβ42‐displaying NVs as a model system with genetically defined surface protein states, it was demonstrated that equivalent‐circuit‐based impedance analysis can quantitatively distinguish membrane protein configurations, providing a standardizable approach applicable to diverse EV surface markers. Through multifaceted analysis spanning ML optimization across broad frequency ranges, equivalent circuit modeling, and mechanistic structure‐based predictions, the vesicle membrane capacitance C_m_ was found to correlate with the oligomeric state of membrane‐bound Aβ, and the change in reactance (Δ|X/X_0_|) at 1 kHz was identified as the optimal parameter for discriminating Aβ oligomeric states within this system. Notably, these analytical conditions, established using gene‐defined NVs, were further validated through time‐dependent monitoring of membrane‐bound Aβ oligomerization, confirming the platform's ability to detect changes in surface protein state.

One of the key findings of this study was that X, rather than |Z| or R, provided the highest sensitivity to membrane protein variations. This can be understood from the impedance relationship, since the equivalent‐circuit analysis showed that membrane capacitance (C_eff_) accounts for less than 6% of the total |Z| at 1 kHz, the capacitive contribution arising from vesicle membrane properties is largely masked by R. In contrast, X extracted from the vesicle‐induced impedance change (ΔZ) could directly reflect the reactive components of the system, thereby isolating the membrane‐associated capacitive signal and increasing sensitivity to changes in surface protein composition. This mechanistic interpretation, derived from the equivalent‐circuit model, is consistent with the ML optimization results, which independently identified |ΔX/X_0_| at 1 kHz as the parameter exhibiting the most reliable correspondence to the genetically defined membrane protein states.

The integration of impedance analysis with ML‐assisted optimization offers significant advantages, including enhanced sensitivity and specificity, as well as the ability to systematically optimize analytical parameters for vesicle surface protein analysis. This approach enables robust discrimination of subtle differences in membrane protein composition without labeling the membrane, a challenge for conventional techniques. Established methods for EV surface protein characterization each face specific constraints. Labeling‐based techniques such as ELISA and Western blotting often require vesicle lysis and sequential target‐specific labeling, and flow cytometry demands fluorescent labeling. Label‐free spectroscopic methods, including SERS and SPR, while capable of multiplexed detection, typically require specialized substrates and complex spectral interpretation. In comparison, the impedance‐based approach presented here enables label‐free detection of membrane protein variations using a straightforward interdigitated microelectrode design, with ML systematically identifying optimal measurement conditions rather than relying on empirical parameter selection. The analytical framework relies on widely adopted ML classifiers and interpretable equivalent‐circuit parameters, facilitating reproducibility across laboratories and lowering barriers to implementation. Furthermore, although Aβ42 was used as a model membrane protein in this study, the methodology may apply to the analysis of other EV surface proteins. By enabling quantitative, intact assessment of surface protein heterogeneity, this platform may support future studies aimed at discovering and characterizing disease‐associated EV markers. To assess the applicability of this analytical framework beyond engineered NVs, a preliminary validation was performed using native EVs isolated from unmodified cells. The reactance‐based analysis at 1 kHz successfully distinguished the membrane protein‐enriched EVs from membrane protein‐disrupted EVs (Figure S7), suggesting that the optimized measurement conditions derived from the NV model system can be extended to biologically relevant EV samples.

While this study focused on identifying a single optimal frequency‐feature pair to maximize mechanistic interpretability through equivalent‐circuit modeling, multi‐parameter classification approaches using AI that combine multiple impedance features across frequencies could potentially achieve high discrimination accuracy. The ML framework developed here is readily extensible to such multi‐feature analyses, and future studies could explore this direction for applications where maximizing classification performance is prioritized over single‐parameter mechanistic interpretation.

Additionally, engineered NVs with membrane proteins controlled at defined copy numbers could serve as reference materials to advance vesicle surface protein analysis techniques. In this study, an antibody recognizing Aβ from monomer to oligomer (6E10) and an oligomer‐specific antibody (A11) were combined to estimate the protein density on the vesicle surface, using amyloid‐β as a model membrane protein. DLS measurements and AlphaFold‐based structural analysis showed that increasing Aβ expression did not significantly change vesicle size, indicating that modulation primarily affected surface protein loading rather than overall vesicle shape. These findings suggest the potential of using controlled protein expression to create membrane protein–rich versus protein–poor vesicle populations that can be distinguished and analyzed separately. Beyond Aβ, this approach may apply to other membrane proteins, enabling applications in EV method development and benchmarking.

The regulation of Aβ oligomeric states using engineered NVs also provides a platform for further studies. The availability of samples with precisely controlled oligomeric structures offers a quantitative framework for studying Aβ aggregation kinetics, allowing systematic investigation of environmental factors and therapeutic candidates that affect oligomer formation and stability. Beyond Aβ, the membrane‐anchored protein display method could potentially be applied to other disease‐related proteins involved in neurodegenerative disorders, such as α‐synuclein in Parkinson's disease or tau in tauopathies.

Despite the comprehensive analyses of Aβ‐NVs and impedance spectroscopy, several limitations should be acknowledged for future development and practical application. The equivalent circuit analysis showed that membrane capacitance (C_eff_) accounted for less than 6% of the total impedance magnitude at the optimal frequency of 1 kHz. While this small contribution was sufficient for discriminating Aβ42 oligomeric states via reactance analysis, further refinement of circuit models could amplify the capacitive signal and enhance detection sensitivity. Additionally, the systematic optimization was limited to logarithmically spaced frequencies and concentrations, leaving unexplored intermediate parameter spaces. High‐resolution frequency impedance analysis may reveal finer details in the impedance response and potentially identify even more sensitive analytical conditions. Finally, the current study employed a single model protein to establish proof of concept; generalizability to other surface proteins with different structural and charge properties remains to be validated for establishing engineered NVs as a general model standard in EV surface protein analysis.

In conclusion, this study introduces a standardizable framework that combines synthetic biology, impedance spectroscopy, machine learning optimization, and equivalent‐circuit‐based mechanistic modeling to address the critical need for quantitative vesicle surface analysis. By demonstrating that engineered nanovesicles can serve as tunable reference standards and that reactance‐based measurements at an ML‐identified optimal frequency can differentiate membrane protein states with mechanistic interpretability, this work establishes foundational principles for developing impedance‐based EV surface protein analysis. The integration of precisely engineered biological samples with data‐driven analytical optimization offers a generalizable strategy applicable to diverse vesicle surface markers, supporting the development of standardized EV‐based diagnostics, protein biophysics research, and precision medicine applications in neurodegenerative diseases.

## 4 Materials and Methods

Details for western blotting, confocal microscopy, immunofluorescence staining, and sandwich ELISA were provided in the Supporting Information (Sections S1.2, S1.3, S1.4, and S1.6).

### 4.1 Materials

All chemicals and materials were used as received and are listed in Table S1, along with instrument and software details.

### 4.2 Plasmid Construction

DNA cloning was performed using standard molecular biology techniques. The TM‐Myc‐mCherry coding sequence was PCR‐amplified from the pSM005 vector [63] and subcloned into the pcDNA3.1(+) expression vector. A GS linker was then fused to the construct, followed by the individual cloning of amyloid beta 42 (Aβ42) monomer, trimer, and nonamer gene sequences to generate different Aβ42 repeats, such as monomer (Aβ42‐M), trimer (Aβ42‐T), and nonamer (Aβ42‐N) plasmids. All recombinant plasmids were sequence‐verified, propagated in E. coli DH5α, and used for subsequent protein expression studies.

### 4.3 Production and Purification of Aβ42 Nanovesicles

HeLa cells were transfected with the pcDNA3.1 vector encoding Aβ42‐M, Aβ42‐T, and Aβ42‐N, individually, using Lipofectamine 3000 according to the manufacturer's instructions. Transfected cells were cultured in Dulbecco's Modified Eagle's Medium (DMEM) supplemented with 10% fetal bovine serum and 1% penicillin–streptomycin at 37°C with 5% CO_2_ until they reached 80%–90% confluency. Cells were washed with phosphate‐buffered saline (PBS), detached, and collected by centrifugation at 1000 x*g* for 5 min. The pellet was resuspended in PBS and sequentially extruded through polycarbonate membranes with pore sizes of 10, 5, and 1 µm (≥10 passes each) using a mini‐extruder system. Ultracentrifugation at 15 000 x*g* for 10 min at 4°C was performed after the 10 µm extrusion and again after the final 1 µm extrusion, and the supernatant containing purified nanovesicles was collected and stored at 4°C for further experiments.

### 4.4 In Silico Prediction of Fusion Protein Structures

The three‐dimensional structures of Aβ42‐M, Aβ42‐T, and Aβ42‐N were generated using ColabFold v1.5.5: AlphaFold2 [46, 47]. The predicted structures were subsequently analyzed and visualized using PyMOL software.

### 4.5 IMEs Fabrication and Functionalization

IMEs were fabricated on glass wafers by a standard lift‐off photolithography process. Each device comprised 42 interdigitated finger pairs (width, 5 µm; gap, 5 µm), formed from a Cr adhesion layer (≈20 nm) and a Pt layer (≈200 nm) deposited by e‐beam evaporation. The fabricated IME chips were integrated with a 20‐µm‐high PDMS microfluidic channel and functionalized with capture antibodies. Briefly, the glass substrate was aminosilanized with (3‐aminopropyl)triethoxysilane (APTES), and the PDMS channel was epoxysilanized with (3‐glycidyloxypropyl)trimethoxysilane (GLYMO), after which the two were aligned and bonded. An anti‐amyloid‐β antibody (clone 6E10) was then immobilized within the microchannel via 1‐ethyl‐3‐(3‐dimethylaminopropyl)carbodiimide (EDC) and N‐hydroxysulfosuccinimide sodium salt (sulfo‐NHS) coupling chemistry. Detailed fabrication and functionalization procedures were provided in the Supporting Information (Section S1.5).

### 4.6 Impedance Measurement of Aβ‐NVs

NVs were quantified by NTA and diluted to the specified concentration with 1× PBS. Using an antibody‐immobilized IME sensor, each sensor was filled with 0.1× PBS, and impedance was measured over the range of 10 Hz to 1 MHz. To mitigate matrix effects, devices were then exposed to 1× PBS for 30 min, rinsed, refilled with 0.1× PBS, and a second baseline impedance was recorded. For NVs detection, the channel was incubated with the NVs for 30 min, rinsed with 0.1× PBS, and a post‐incubation impedance was measured.

From the measured impedance and phase, resistance (R) and reactance (X) were calculated according to the following Equations (14) and (15):

(14) $$R=\left|Z\right|\;\cos\theta$$

(15) $$X=\left|Z\right|\;\sin\theta$$

The relative changes induced by NVs were calculated as follows for impedance, and the same calculation scheme was applied for resistance and reactance, where changes caused by 1×PBS treatment were considered as the baseline according to the following Equation (16):

(16) $$\begin{array}{c} \left|\frac{Z}{Z_{0}}\right|\left(\%\right)=\left(\frac{Z_{NVs}-Z_{Antibody}}{Z_{Antibody}}\right)-\left(\frac{Z_{1XPBS}-Z_{Antibody}}{Z_{Antibody}}\right)\left(\%\right) \end{array}$$

here, Z_Antibody_ is the impedance after antibody immobilization, Z_1XPBS_ is the impedance after 30 min incubation with 1×PBS, and Z_NVs_ is the impedance after NV treatment.

### 4.7 Machine‐Learning‐Guided Optimization

To identify the optimal analytical condition for the IME biosensor, we systematically evaluated 18 frequency‐feature combinations, comprising three impedance features (Δ|Z/Z_0_|, Δ|R/R_0_|, Δ|X/X_0_|) measured at six frequencies spanning 10 Hz to 1 MHz (10 Hz, 100 Hz, 1 kHz, 10 kHz, 100 kHz, 1 MHz). The dataset consisted of impedance measurements from n = 12 independent replicates for each of the four vesicle types (C‐NV, M‐NV, T‐NV, N‐NV) across six concentration levels (10^4^, 10^5^, 10^6^, 10^7^, 10^8^ particles mL^−1^). For each frequency‐feature combination, performance was quantified using three complementary metrics normalized to a 0–1 scale: linearity score (Score_Linearity_), order score (Score_Order_), and classification score (Score_Classification_). The linearity score was calculated as the R^2^ value from semi‐logarithmic regression (log_10_ concentration vs. response) across the entire concentration range (10^4^–10^8^ particles mL^−1^), evaluating the calibration quality of the biosensor. The order score was computed as Spearman's rank correlation coefficient between the mean impedance response across all concentrations and the known biological order of membrane‐bound Aβ42 peptides (0, 1, 3, 9 copies for C‐NV, M‐NV, T‐NV, N‐NV, respectively), assessing whether the sensor response monotonically reflects Aβ42 content. The classification score evaluated the ability of machine learning to discriminate among the four vesicle types. Six classifiers implemented in scikit‐learn were employed: Extra Trees, Random Forest, Gradient Boosting, Support Vector Machine, Logistic Regression, and k‐Nearest Neighbors. For each concentration level, stratified 5‐fold cross‐validation was performed, and the classification score for each fold was calculated as the average of accuracy and F1‐score:

$$\mathrm{Scor}e_{\mathrm{Clas}\mathrm{sifi}\mathrm{cati}\mathrm{on}}=\frac{\mathrm{Accu}\mathrm{racy}+F1}{2}$$

The final classification score for each frequency‐feature combination was obtained by averaging across all concentration levels and all six classifiers. The three individual scores were combined into a comprehensive score (Score_Comprehensive_) using a weighted mean as described in Equation (1), where W_1_, W_2_, and W_3_ represent the relative weights assigned to linearity, order, and classification objectives, respectively. All analyses were performed using Python 3.10.9 with scikit‐learn 1.0.2 on a Mac Studio (2022, Apple M1 Max, 32 GB RAM, macOS Sequoia 15.4.1), with a total computation time of approximately 2–3 min for the complete evaluation. Complete code was provided in Supporting Information: S3.

### 4.8 Soluble Aβ Incubation on Aβ‐NVs

To evaluate the optimized IME sensor, time‐resolved samples were prepared by growing Aβ on Aβ‐NVs. The stability of the vesicles in suspension was verified before incubation through assessment of potential vesicle–vesicle hybridization. Vesicles maintained in 1× PBS at 4°C showed no evidence of aggregation or fusion, confirming compatibility of incubation at this temperature (Figure S5). For peptide growth on the vesicle membrane, Aβ‐NV stocks were first equalized in particle concentration using NTA and diluted in 1× PBS to 2×10^10^ particles mL^−1^ (100 µL). Simultaneously, soluble Aβ42 peptide was prepared at 2 µM in 1× PBS (100 µL). Both solutions were mixed in a 1:1 ratio to produce a final mixture of 200 µL containing 1×10^10^ particles mL^−1^ of Aβ‐NVs and 1 µM Aβ42. Incubations were conducted at 4°C for 0, 6, and 24 h. Following each time point, the unbound peptide was removed via ultracentrifugation at 100 000× *g* for 60 min at 4°C, performed twice. Vesicle pellets were then gently resuspended in 1× PBS and re‐quantified by NTA for subsequent measurements.

### 4.9 Statistical Analysis

All quantitative data are presented as mean ± standard deviation (SD). The number of independent replicates (n) for each experiment is specified in the corresponding figure legends: impedance measurements were performed with n = 12 independent replicates per condition (Figures 3, 4, 6, 8), zeta potential and DLS measurements for characterization of NVs performed with n = 4 per group (Figure 2), DLS measurement for stability assessment of NVs performed with n = 5 per group (Figure S5). Sandwich ELISA assays with n = 3 per concentration, and lung cancer cell (H1437) derived EV measured with impedance with n = 31 independent replicates per condition (Figure S7). Dose‐response relationships were evaluated by semi‐logarithmic linear regression, and goodness‐of‐fit was reported as the coefficient of determination (R^2^). Comparisons among the four nanovesicle types (C‐NV, M‐NV, T‐NV, N‐NV) were performed using ordinary one‐way analysis of variance (ANOVA) followed by Tukey's multiple comparison post‐hoc test. Statistical significance was defined as follows: ns (not significant, p ≥ 0.05), *p < 0.05, **p < 0.01, ***p < 0.001, and ****p < 0.0001. All statistical analyses and curve fitting were performed using GraphPad Prism 10.1.1 (GraphPad Software, San Diego, CA, USA).

## Author Contributions

**Jaeyoon Song**: investigation, validation, formal analysis, visualization, software, writing – original draft, writing – review and editing, methodology. **Sehyeon Kim**: investigation, validation, formal analysis. **Jinsik Kim**: conceptualization, methodology, supervision, funding acquisition, writing – original draft, writing – review and editing, data curation. **Neethu Ramakrishnan**: methodology, investigation, validation, formal analysis, writing – original draft. **Huiseop Lee**: investigation, validation, formal analysis. **Youngeun Kwon**: conceptualization, supervision, methodology, writing – review and editing, writing – original draft.

## Conflicts of Interest

The authors declare no conflicts of interest.

## Supporting information




**Supporting File 1**: advs77452‐sup‐0001‐SuppMat.docx.

**Supporting File 2**: advs77452‐sup‐0002‐SuppMat.ipynb.

## Data Availability

The data supporting the findings of this study are available from the corresponding author upon reasonable request. The source code for machine‐learning‐guided optimization is included in the Supporting Information.
