# Supplementary material for: Machine‐Learning‐Assisted Impedance Component Analysis Enables Standardizable Surface Protein Analysis of Extracellular Vesicles Using Engineered Nanovesicles
Source: Adv Sci (Weinh). 2026 Aug 29;13(56):e77452. doi: 10.1002/advs.77452 (PMC13525417; doi:10.1002/advs.77452)
Supplement: Supplementary file 1 [file ADVS-13-e77452-s002.docx]

**Supporting Information**

Machine-learning-assisted Impedance Component Analysis enables Standardizable Surface Protein Analysis of Extracellular Vesicles using Engineered Nanovesicles

Jaeyoon Song, Neethu Ramakrishnan, Sehyeon Kim, Huiseop Lee, Youngeun Kwon*, Jinsik Kim*

Department of Biomedical Engineering, College of Life Science and Biotechnology, Dongguk University, Seoul 04620, Republic of Korea

*E-mail: Youngeun Kwon [ykwon@dongguk.edu](mailto:ykwon@dongguk.edu); Jinsik Kim [lookup2@dongguk.edu](mailto:lookup2@dongguk.edu)

**S1. Supplementary materials and methods**

**S1.1 Reagents, instruments, and software**

| **Category** | **Name** | **Supplier** |
| --- | --- | --- |
| Reagents | DNA primers | Bionics (Seoul, Republic of Korea) |
|  | Plasmid kits | Elpis Biotech (Daejung, Republic of Korea) |
|  | Restriction enzymes | New England Biolabs (MA, USA) |
|  | PCR kit | RBC (Taipei, Taiwan) |
|  | Gel extraction kit |  |
|  | Cell-culture reagents | Welgene (Daegu, Republic of Korea)  Thermo Fisher Scientific (MA, USA) |
|  | Lipofectamine^TM^ 3000 | Invitrogen (Carlsbad, USA) |
|  | Bovine serum albumin | KOMA BIOTECH (Republic of Korea) |
|  | Chemiluminescent substrate | Thermo Fisher Scientific (MA, USA) |
|  | streptavidin–horseradish peroxidase |  |
|  | biotinylated detection antibody  (anti-human IgG) |  |
|  | anti-β-amyloid antibody (clone 6E10) | Biolegend (USA) |
|  | anti-amyloid oligomers antibody (clone A11) | Abcam (UK) |
|  | Anti-mCherry antibody |  |
|  | Goat anti-rabbit IgG Fc Alexa Fluor 488 |  |
|  | Aβ42 peptide | Tocris Bioscience (UK) |
|  | Lift-off resist APOL-LO 3202 | KemLab Inc (MA, USA) |
|  | AZ-300MIF developer | Merck (Germany) |
|  | (3-aminopropyl)triethoxysilane | Sigma-Aldrich (USA) |
|  | (3-glycidyloxypropyl)trimethoxysilane |  |
|  | 1-ethyl-3-(3-dimethylaminopropyl)carbodiimide | Tokyo Chemical Industry (Japan) |
|  | N-hydroxysulfosuccinimide sodium salt |  |
|  | Phosphate-buffered saline (1X) | Gibco (USA) |
| Instruments | Extruder system | Avanti Polar Lipids (USA) |
|  | Zetasizer Nano ZS90 | Malvern Panalytical (UK) |
|  | Nanoparticles Tracking Analysis LM10 |  |
|  | ImageQuant LAS 500 | GE Healthcare (USA) |
|  | K1-Fluo Confocal Laser Scanning Microscope | Nanoscope Systems (Republic of Korea) |
|  | Plasma cleaner PS-200 | PLASOL  (Republic of Korea) |
|  | LCR meter IM3536 | Hioki (Japan) |
|  | Ultracentrifuge Optima XE-100 | Beckman Coulter (USA) |
|  | Plate reader Infinite M Plex | Tecan (Männedorf. Switzerland) |
| Software | Python 3.10.9 | - |
|  | Visual Studio Code 1.100.3 | Microsoft (USA) |
|  | ColabFold v1.5.5 | Google (USA) |
|  | PyMOL | Schrödinger (NY, USA) |

**Table S1.** Reagents, instruments, and software used in this study.

**S1.2 Western blotting**

HeLa cells (2×10⁶) were cultured in 100-mm dishes and transfected with plasmids encoding Aβ42-M, Aβ42-T, and Aβ42-N, respectively. Following transfection, cells were incubated at 37°C for 24 h to allow protein expression. Cells were then harvested and lysed using RIPA buffer supplemented with 1 mM protease inhibitor. The resulting lysates were analyzed by western blotting analysis using anti-mCherry primary antibodies followed by goat anti-rabbit IgG conjugated to HRP. Chemiluminescent signals were visualized using the SuperSignal™ ELISA Pico substrate and imaged with an ImageQuant™ LAS 500 system.

**S1.3 Confocal microscopy**

HeLa cells were seeded at a density of 1×10⁵ cells per 35 mm confocal dish and transfected with 2 μg of plasmid DNA encoding Aβ42-M, Aβ42-T, and Aβ42-N, individually, using Lipofectamine™ 3000. Following a 24-hour incubation, cells were imaged using confocal microscopy. mCherry fluorescence was excited at 560 nm, and emission was collected through a 607/75 nm filter. All images were captured using identical laser settings.

**S1.4 Immunofluorescence staining**

HeLa cells expressing Aβ42-M, Aβ42-T, and Aβ42-N were cultured on glass coverslips and fixed individually with 4% (w/v) paraformaldehyde in PBS for 15 min at room temperature. Following fixation, the cells were washed with PBS and blocked with 3% BSA in PBS for 1 h at room temperature to minimize non-specific binding. Subsequently, the cells were incubated overnight at 4°C with rabbit monoclonal anti-β-amyloid 1-42 antibody. After washing with PBS, the cells were incubated for 1 h at room temperature with goat anti-rabbit IgG Fc conjugated to Alexa Fluor® 488 (secondary antibody). The Alexa Fluor® 488 fluorophore was excited at 495 nm, and emission was detected at 519 nm. Finally, the stained cells were thoroughly washed with PBS and imaged using a confocal laser scanning microscope.

**S1.5 IMEs fabrication and functionalization**

A standard lift-off photolithography process fabricated interdigitated microelectrodes (IMEs). Briefly, 0.5-mm-thick 6-inch glass wafers were solvent-cleaned, coated with a bilayer of lift-off resist and positive photoresist, exposed through a chrome mask, and developed to form the IME pattern. A Cr adhesion layer (≈20 nm) and a Pt layer (≈200 nm) were deposited by e-beam evaporation, followed by lift-off to define the metal features. Each device comprised 42 interdigitated finger pairs with a finger width of 5 µm and a 5 µm gap. The fabricated IME chips were integrated with a PDMS microfluidic channel and functionalized with capture antibodies. Glass substrates were first activated by oxygen plasma (50 W, 50 s, 50 sccm O_2_), then aminosilanized with APTES (1% v/v in ethanol) at 25 °C for 2 h 30 min, followed by ethanol rinsing to remove residual silane. A 20-µm-high PDMS channel was molded, treated under the same oxygen-plasma conditions, and epoxysilanized with GLYMO (1% v/v in ethanol) at 25 °C for 2 h 30 min, followed by ethanol rinsing. The PDMS layer was aligned and bonded onto the APTES-modified glass and cured on a hot plate at 130 °C for 20 min. For antibody immobilization, an anti-amyloid-β antibody (clone 6E10) was coupled via EDC and sulfo-NHS chemistry. A solution containing 10 µg mL^-1^ antibody, 2 mM EDC, and 8 mM sulfo-NHS in 1× PBS was introduced into the microchannel and incubated for 2 h 30 min at 25 °C. After reaction, the devices were rinsed sequentially with 1× PBS and deionized water to remove residual reagents. Functionalized sensors were used immediately after preparation.

**S1.6 Sandwich ELISA**

A 96-well plate was used to evaluate the quality of incubated NVs by sandwich ELISA. Wells were coated overnight at 4 °C with 100 µL of capture antibody. Plates were washed twice with PBS containing 0.05% Tween-20 (PBS-T) and once with PBS, then blocked with 200 µL of 1% (w/v) BSA for one hour at 25 °C. After washing as above, 100 µL of NVs was added to each well and incubated overnight at 4 °C with gentle shaking (200 rpm). Wells were washed, followed by incubation with 100 µL of biotinylated detection antibody (0.5 µg mL^-1^ ) for 2h at 25 °C with gentle shaking. After washing, 100 µL of streptavidin–horseradish peroxidase (0.5 µg mL^-1^ ) was added and incubated for 1 h at 25 °C. Finally, 100 µL of TMB substrate was added, and the reaction was developed for 5 min at 25 °C in the dark, and absorbance was read at 450 nm using a microplate reader.

**S2. Equivalent-circuit derivation.**

**S2.1 Baseline circuit and notation**

An IME biosensor in 0.1×PBS is represented by a solution branch Z_s_ (parallel R_s_ || C_s_) in series with two double-layer capacitances C_dl_:

$Z_{s}=\frac{R_{s}}{1+j\omega R_{s}C_{s}}$	(S1)

$Z_{A}=\frac{2}{j\omega C_{dl}}+Z_{s}$	(S2)

**S2.2 Circuit with vesicles**

When Aβ-NVs bridge the field lines, a vesicle branch Z_ves_ is placed in parallel with Z_s_:

$Z_{B}=\frac{2}{j\omega C_{dl}}+\frac{Z_{s}Z_{ves}}{Z_{s}+Z_{ves}}$	(S3)

The vesicle branch comprises an internal (lumen) resistance R_l_ in series with a membrane element allowing non-ideal capacitance:

$Z_{ves}=R_{l}+({CPE}_{m}|\left| R_{m} \right), {CPE}_{m}=\frac{1}{Q(j\omega)^{\alpha}} (0<\alpha\leq1)$	(S4)

Here R_m_ is the membrane leak; Q, α are constant-phase-element (CPE) parameters. The ideal-capacitor limit is $\alpha=1, Q=C_{m}$and $R_{m}\to\infty$.

**S2.3 Exact impedance change**

The change upon vesicle immobilization is

$\Delta Z\equiv Z_{B}-Z_{A}=\frac{Z_{s}Z_{ves}}{Z_{s}+Z_{ves}}-Z_{s}=-\frac{Z_{s}^{2}}{Z_{s}+Z_{ves}}$	(S5)

Introducing the vesicle admittance $Y_{ves}=1/{Z_{ves}}$ and using the baseline Z_A_ gives a compact form of equation (4), as described in the manuscript.

$\Delta Z=-\frac{Z_{A}^{2}Y_{ves}}{1+Z_{A}Y_{ves}}$	(4)

**S2.4 Exact separation into** $\boldsymbol{\Delta R}$ **and** $\boldsymbol{\Delta X}$

Let $Z_{A}=R_{A}+jX_{A}$and $Y_{ves}=G+jB$ with real conductance $G(\omega)$and $B(\omega)$. Define

$1+Z_{A}Y_{ves}=D_{R}+jD_{I}$	(S6)

$D_{R}=1+R_{A}G-X_{A}B$, $D_{I}=R_{A}B+X_{A}G$	(S7)

and

$Z_{A}^{2}Y_{ves}=N_{R}+jN_{I}$	(S8)

$N_{R}=\left( R_{A}^{2}-X_{A}^{2} \right)G-2R_{A}X_{A}B$, $N_{I}=\left( R_{A}^{2}-X_{A}^{2} \right)B+2R_{A}X_{A}G$	(S9)

Then

$\Delta R=-\frac{N_{R}D_{R}+N_{I}D_{I}}{D_{R}^{2}+D_{I}^{2}}$	(S10)

$\Delta X=-\frac{N_{I}D_{R}-N_{R}D_{I}}{D_{R}^{2}+D_{I}^{2}}$	(S11)

These identities hold for the full CPE_m_ ||R_m_ membrane and show that $\Delta R$ and $\Delta X$ are weighted combinations of G (luminal path) and B (membrane capacitance), modulated by the baseline Z_A_.

**S2.5 Coupling magnitude and mid-band approximation**

The denominator contains the dimensionless coupling magnitude

${\mu\equiv|Z}_{A}Y_{ves}|$	(5)

This quantity measures how strongly the vesicle branch perturbs the baseline circuit. In practice, $\mu$ computed for each chip from the fitted parameters was found to be well below unity in the analysis window; in particular, $\mu\leq0.3$between 100 Hz and 10 kHz for most chips (**Fig. S2C**). Under weak-coupling condition ($\mu\ll1)$, $(1+Z_{A}Y_{ves})^{-1}\approx1$, and equation (4) reduces to

$\Delta Z\approx-Z_{A}^{2}Y_{ves}$	(6)

so that the observed change is essentially the vesicle admittance, scaled and rotated by the baseline.

**S2.6 R_l_–C_m_ simplification for intuition** (**Figure 5C–D**)

For interpretability, the membrane is idealized $(\alpha=1, R_{m}\to\infty)$:

$Z_{ves}=R_{l}+\frac{1}{j\omega C_{m}}$	(7)

$Y_{ves}=\frac{1}{R_{l}+(1/{j\omega C_{m})}}=\frac{R_{l}}{R_{l}^{2}+(1/{\omega C_{m}})^{2}}+j\frac{1/{\omega C_{m}}}{R_{l}^{2}+(1/{\omega C_{m}})^{2}}$	(8)

After removing baseline-dependent gains, the sensitivities follow

$\Delta R\propto\frac{R_{l}}{R_{l}^{2}+(1/{\omega C_{m}})^{2}}$	(9)

$\Delta X\propto\frac{1/{\omega C_{m}}}{R_{l}^{2}+(1/{\omega C_{m}})^{2}}$	(10)

Thus, $\Delta R$ is governed by the access resistance R_l_ (vesicle concentration, size), whereas $\Delta X$ is governed by the membrane charging term C_m_ (dielectric properties and protein content of membrane).

**Figure S1.** Comprehensive-score heatmaps under different weighting schemes. (A) Linearity-weighted evaluation with weights W_1_:W_2_:W_3_ = 0.6:0.2:0.2 (prioritizing sensor’s detection quality). The highest Score_Comprehensive_ was achieved for reactance (Δ|X/X_0_|) at 1 kHz (asterisk). (B) Order-weighted evaluation with weights W_1_:W_2_:W_3_ = 0.2:0.6:0.2 (prioritizing monotonic correspondence to Aβ42 copy number). The optimal condition shifted to reactance (Δ|X/X_0_|) at 1 kHz (asterisk), consistent with the strictly monotonic response shown in Figure 4H. (C) Classification-weighted evaluation with weights W_1_:W_2_:W_3_ = 0.2:0.2:0.6. The optimal condition returned to impedance magnitude (Δ|Z/Z_0_|) at 1 kHz (asterisk).

The stability of the machine learning-guided optimization method was tested by systematically changing the relative weights given to the three objective functions in the comprehensive scoring algorithm. (A) Linearity-weighted evaluation (weights: linearity 0.6, order 0.2, classification 0.2) identified 1 kHz reactance change (Δ|X/X_0_|) as the optimal frequency-parameter combination (indicated by an asterisk). (B) Order-weighted evaluation (weights: linearity 0.2, order 0.6, classification 0.2) identified 1 kHz reactance change (Δ|X/X_0_|) as optimal, which matches the monotonic response to Aβ copy number shown in Fig. 4H. (C) Classification-weighted evaluation (weights: linearity 0.2, order 0.2, classification 0.6) returned to impedance magnitude (Δ|Z/Z_0_|) at 1 kHz as the best parameter. Despite the three-fold variation in objective weights, the optimal frequency converged to 1 kHz in all weighting schemes. This consistent convergence to the 1 kHz operating point, regardless of the weighting strategy, was indicative of the robustness of the ML-guided approach and suggests that this frequency is a fundamental feature of the impedance analysis of vesicles.

**Figure S2.** Coupling magnitude and crossover frequency from equivalent circuit fit. (A) Frequency dependence of the coupling magnitude. Across chips, $\mu$ remained below unity throughout 100 Hz–10 kHz and was typically <0.3 between 100 Hz and 1 kHz, validating the weak-coupling approximation used for interpretation. (n = 12 for each frequency) (B) Distribution of crossover frequencies f_c_ (left axis) defined as the frequency at which the vesicle-branch susceptance equals its conductance, |B(f_c_)|=G(f_c_), computed from the chip-wise circuit fits. The histogram is overlaid with the ML-derived comprehensive score versus frequency (right axis). f_c_ values cluster near 1 kHz, in agreement with the ML result that 1 kHz is the optimal single-feature condition. (n = 30 for f_c_ distribution)

Coupling magnitude $\mu$ computed for each chip from the fitted parameters was found to be well below unity in the analysis window; in particular, $\mu\leq0.3$ between 100 Hz and 10 kHz for most chips (**Figure S2A**). Under this weak-coupling condition, $(1+Z_{A}Y_{ves})^{-1}\approx1$, and equation (4) can be reduced to $\Delta Z\approx-Z_{A}^{2}Y_{ves}$, so that the observed change is essentially the vesicle admittance, scaled and rotated by the baseline.

The crossover frequency, fc, is defined as the frequency at which the susceptive and conductive components of the vesicle-branch admittance are equal in magnitude. The defined f_c_ satisfies

$|B(f_{c})|=G(f_{c})$	(S12)

Under the small-perturbation regime ($\mu\left( f \right)=|Z_{A}\left( f \right)Y_{ves}\left( f \right)|\ll1$), this equality marks the point where the vesicle branch provides comparable resistive and capacitive admittances, so that changes in resistance and reactance contribute similarly to the measured impedance perturbation.

Chip-wise f_c_ inferred from the circuit fits cluster around 1 kHz, indicating the frequency range where the vesicle branch offers similar conductance and susceptance (**Figure S2B**). This distribution matches the ML analysis that identified 1 kHz reactance as the most informative single feature for both concentration calibration and Aβ copy-number ordering. The ML-derived comprehensive score was computed by averaging the three features for each concentration, using a balanced 1:1:1 weighting scheme (**Figure 3B**).

**Figure S3**. Structure-based prediction of membrane-anchored protein orientation and capacitance. (A) Five structural models generated by AlphaFold2 for each Aβ42 variant, spanning from the mCherry N-terminus (blue, anchoring point to membrane-proximal Myc tag) to the C-terminal Aβ42 repeats (red). Models shown: Aβ42-M (m1–m5), Aβ42-T (t1–t5), and Aβ42-N (n1–n5). (B) Schematic illustration of the membrane docking procedure. The TM and Myc tag were constrained perpendicular to the plasma membrane, with the first mCherry residue serving as a fixed anchor point. Each AlphaFold2-generated structure was systematically rotated in 2° increments along the x- and y-axes to identify optimal poses satisfying two criteria: (i) no membrane penetration and (ii) protrusion heights consistent with DLS measurements. (C) Predicted extrusion heights for each structural model after pose optimization. Shaded regions indicate DLS-derived target height ranges: Aβ42-M, 3.5 ± 1.0 nm; Aβ42-T, 5.0 ± 0.9 nm; Aβ42-N, 5.5 ± 0.95 nm. Two Aβ42-N models failed to achieve non-penetrating poses within the target range and were excluded from analysis. Structures with optimal height agreement (yellow borders) were selected for capacitance modeling. (D) Protein-specific capacitance values calculated for the selected optimal poses using parallel-plate capacitor approximation. The capacitance was computed from the effective projected area and protein height (thickness of dielectric layer).

To determine the effective capacitance contribution of membrane-displayed Aβ42 proteins, we performed systematic structural modeling and orientation optimization. Five structural models for each Aβ42 variant (Aβ42-M, Aβ42-T, and Aβ42-N) were generated using AlphaFold2 (**Figure S3A**). The TM domain and Myc tag were assumed to be perpendicular to the plasma membrane, while the extracellular mCherry-Aβ42 chain adopted various conformations. The first residue of mCherry (shown in blue) served as the anchoring point to the membrane-proximal Myc tag terminus.

Each generated structure was anchored at the Myc tag position, consistent with the genetic architecture of the fusion constructs (**Figure S3B**). To identify physiologically relevant orientations, we performed systematic rotational sampling at 2-degree increments along the x- and y-axes, optimizing for two criteria: (i) prevention of membrane penetration and (ii) achievement of appropriate protrusion heights. Target protrusion heights were derived from DLS measurements (**Figure 1F**), assuming symmetric bilateral protein display on vesicles: 3.5 ± 1.0 nm for Aβ42-M, 5.0 ± 0.9 nm for Aβ42-T, and 5.5 ± 0.95 nm for Aβ42-N.

The optimal pose for each structural model was selected based on the closest agreement with DLS-predicted height ranges (**Figure S3C**, shaded regions). The poses with the best agreement (yellow borders) were used for subsequent capacitance calculations.

Next, protein-specific capacitance values were calculated using the effective area and height of each optimized pose, treating the protein layer as a parallel-plate capacitor (**Figure S3D**). As expected, Aβ42-N, containing the highest number of Aβ repeats, exhibited the greatest capacitance contribution, consistent with its more extended and charge-dense structure.

**Figure S4**. The contribution of C_eff_ to the total impedance Z_B._ The C_eff_ fraction was determined across the entire frequency range to assess the contribution of C_eff_ to the impedance signal Z_B_ after vesicle capture. To ensure comparability across samples at the same particle concentration, the contribution was calculated at 10^6^ particles mL^-1^ for all samples. Since C_m_ was fitted as CPE_m_ in the equivalent circuit fitting procedure, C_eff_ values were obtained at each individual frequency, and the C_eff_ fraction was subsequently derived as the ratio of C_eff_ to Z_B_.

The calculated contribution rates were predominantly below 10% across all frequency ranges for all vesicle types examined (C, M, T, and N). This indicates that the capacitance signal originating from the captured vesicles represents a relatively small fraction of the total circuit impedance. These results highlight the importance of capacitance-centered analysis rather than total impedance analysis for accurate vesicle characterization, as the dominant contributions from other circuit elements would otherwise obscure the vesicle-specific signal.

**Figure S5.** Stability of NVs during incubation in the absence of soluble Aβ. (A) Size monitoring at 4°C over 168 hours showing no significant changes in hydrodynamic diameter for any vesicle type. Data represent mean ± SD (n = 5 for each time point). (B) Size monitoring at 37° C reveals progressive increases in hydrodynamic diameter for all vesicle types beginning after 24 hours, indicating vesicle hybridization and fusion. Data represent mean ± SD (n = 5 for each time point). (C) Representative cryo-TEM images of hybridized vesicles. Scale bars: 100 nm (upper) and 50 nm (lower).

Prior to inducing the growth of soluble Aβ on Aβ-NVs, the potential for hybridization between Aβ-NVs at different incubation temperatures was assessed. To evaluate the stability of nanovesicles under incubation conditions, C-NVs (control vesicles to examine inherent NV hybridization), M-NVs, and N-NVs (to assess the effect of membrane-bound Aβ) were incubated and monitored for size changes over a period of up to 168 hours (0, 2, 8, 24, 72, 120, and 168 h), with finer temporal resolution at early time points to capture any rapid changes within the first 24 hours.

At 4°C, no significant size changes were observed for any of the vesicle types during the entire 168-hour monitoring period, with fold changes remaining near 1.0 at all time points (**Figure S5A**). In contrast, at 37°C, all vesicle types showed progressive size increases that became apparent after 24 hours and reached fold changes of approximately 2.0 by 96-120 hours (**Figure S5B**). The occurrence of vesicle hybridization at 37°C was further confirmed through cryo-TEM imaging, which revealed morphological evidence of fused vesicular structures (**Figure S5C**).

These results established that incubation at 4°C effectively suppresses hybridization between nanovesicles for at least 168 hours, while permitting selective aggregation between membrane-bound Aβ42 and soluble Aβ42 species. This temperature-dependent behavior provides a controlled experimental framework for studying Aβ aggregation dynamics on vesicle membranes without the confounding effects of vesicle-vesicle fusion.

**Figure S6.** Oligomer-specific ELISA (A11) confirms time-dependent oligomerization of NVs. (A) A11 sELISA of vesicles without soluble Aβ incubation (0 h). C-NVs and M-NVs were not detected, while T-NVs and N-NVs exhibited semi-logarithmic linear responses, with N-NVs showing higher signal intensity than T-NVs. Linear regression R^2^ values: C-NV, 0.00; M-NV, 0.86; T-NV, 0.97; N-NV, 0.96. Data represent mean ± SD (n = 3 for each concentration). (B) A11 sELISA after 24 h incubation with soluble Aβ42. C-NVs remained undetectable, while M-NVs, T-NVs, and N-NVs converged to comparable signal intensities with semi-logarithmic linear responses. Linear regression R^2^ values: C-NV, 0.01; M-NV, 0.97; T-NV, 0.95; N-NV, 0.98. Data represent mean ± SD (n = 3 for each concentration). (C) Direct comparison of C-NVs and M-NVs before (0 h) and after (24 h) incubation. C-NVs showed no A11 reactivity at either time point, confirming the absence of oligomeric Aβ and preventing non-specific aggregation of soluble Aβ42. M-NVs exhibited a clear transition from A11-negative (0 h) to A11-positive (24 h), demonstrating conformational maturation from monomeric to oligomeric Aβ states. Data replotted from panels A and B.

To complement the 6E10-based characterization and further assess the conformational changes in Aβ -NVs during incubation, sELISA was performed using A11, an antibody specific for oligomeric Aβ species. Prior to incubation, the A11 sELISA showed selective detection patterns, with C-NVs and M-NVs not captured by the anti-oligomer antibody, while T-NVs and N-NVs showed positive signals (Figure S6A). This initial profile aligned with the presence of oligomeric Aβ structures on T-NVs and N-NVs, whereas M-NVs contained monomeric Aβ species at this stage.

After a 24-hour incubation at 4°C, a noticeable change in the detection pattern was observed. M-NVs became detectable by A11 sELISA, and the signal intensities for M-NVs, T-NVs, and N-NVs aligned to similar levels (**Figure S6B**). This change suggested that the membrane-bound Aβ on M-NVs matured into oligomeric conformations during incubation. Importantly, C-NVs remained undetectable throughout, indicating that the soluble Aβ added during incubation specifically aggregated onto membrane-bound Aβ rather than forming independently or attaching to control vesicles without Aβ.

The comparison between M-NVs at 0 hours and 24 hours clearly demonstrates this conformational transition from monomeric to oligomeric Aβ states (**Figure S6C**). These results confirm that physiologically-formed oligomeric Aβ -NVs were successfully generated through the incubation process, representing oligomer species that develop under natural conditions rather than the gene-defined oligomers directly expressed on N-NVs.

**Figure S7.** Reactance-based discrimination of native EVs with different membrane protein densities on the IME biosensor. (A) Schematic illustration of the two native EV types used for validation. EVs were isolated from conditioned media of H1437 lung cancer cells using two separation methods that differ in cargo preservation: microfluidics-separated EVs (MEVs), which retain a higher proportion of surface proteins and other cargo, and ultrafiltration-separated EVs (UEVs), in which cargo preservation is reduced. Reactance-based impedance analysis was then performed to determine whether the platform could distinguish EVs differing in surface protein content. (B-D) Dose-response curves at 1 kHz for (B) Δ|Z/Z_0_|, (C) Δ|R/R_0_|, and (D) Δ|X/X_0_|. All features exhibited semi-logarithmic linear responses across 10^3^–10^7^ particles ml^-1^. Linear regression R^2^ values for UEVs and MEVs were: (B) 0.89, 0.69; (C) 0.73, 0.65; (D) 0.89, 0.85, respectively. Data represent mean ± SD (n = 31 for each concentration). (E-G) Direct comparison of impedance features between UEVs and MEVs at 10^5^ particles ml^-1^. (E) |ΔZ/Z_0_| showed no significant difference (ns; two-tailed unpaired t-test, t = 1.973, df = 60, p = 0.05). (F) |ΔR/R_0_| showed no significant difference (ns; t = 0.7962, df = 60, p = 0.43). (G) |ΔX/X_0_| exhibited a highly significant difference between the two groups (****p < 0.0001; t = 5.303, df = 60). Data represent mean ± SD (n = 31 per group). (H) Schematic of the sandwich fluorescence verification assay, in which EVs captured on the anti-CD63-functionalized IME sensor surface were labeled with Alexa 488-conjugated anti-CD63 (MEM-259) to confirm surface capture (upper panel). Representative fluorescence images (lower panel) show green fluorescent signals from antibody-labeled EVs immobilized between the IMEs. Fluorescence intensity was quantified as the mean intensity over a 100 × 150 μm sensing area using ImageJ. (I) Fluorescence intensity as a function of EV concentration for UEVs and MEVs. Both EVs shows weak semi-logarithmic linear trend across 10^3^–10^7^ particles ml^-1^. Data represent mean ± SD (n =9 for each concentration). (J) Comparison of fluorescence intensities between UEVs and MEVs immobilized surface, confirming that comparable amounts of EVs were captured on the sensor surface (ns, p = 0.94), indicating that the reactance difference observed in (G) arises from differences in membrane protein composition rather than differential EV capture efficiency.

To evaluate whether the reactance-based surface protein analysis proposed using engineered NVs on the IME biosensor is applicable to native EVs, a validation study was conducted using two types of EVs differing in surface protein content. In our recent study^[2]^, we reported that EVs isolated from the same cell source exhibit different levels of surface protein preservation depending on the separation method. Based on these findings, the same EV types characterized in that study were used here to assess whether the reactance-based analysis can effectively discriminate differences in surface protein abundance on native EVs.

Two types of EVs were prepared from H1437 lung cancer cells (**Figure S7A**): microfluidics-separated EVs (MEVs), which retain surface proteins in a well-preserved state, and ultrafiltration-separated EVs (UEVs), in which surface proteins are relatively less preserved due to the dead-end filtration mechanism. Because both EV types originate from the same parental cell line, they share a common cargo composition, differing primarily in the quantity of preserved surface proteins. This makes them an optimal sample pair for isolating the effect of surface protein abundance on impedance measurements. All EV samples were prepared following the identical protocols described in the reported^[2]^.

Impedance measurements were performed on the anti-CD63 (TS63 clone)-functionalized IME biosensor at 1 kHz, the optimal frequency identified through ML optimization, across concentrations ranging from 10^3^ to 10^7^ particles mL^-1^ (**Figure S7B–D**). All three impedance features (|ΔZ/Z_0_|, |ΔR/R_0_|, and |ΔX/X_0_|) exhibited semi-logarithmic linear trends, confirming dose-dependent responses consistent with those observed for engineered NVs. To determine whether the two EV groups could be discriminated, a direct comparison was performed at 10^5^ particles mL^-1^, the midpoint of the measured concentration range. While |ΔZ/Z_0_| and |ΔR/R_0_| showed no significant differences between UEVs and MEVs (**Figure S7E–F**), |ΔX/X_0_| exhibited a highly significant difference (p < 0.0001; **Figure S7G**). These results confirm that reactance analysis at 1 kHz provides the most surface protein-sensitive discrimination among the impedance features, validating the analytical conditions derived from the engineered NV model system and demonstrating their applicability to native EV surface protein research.

To further verify that the observed reactance difference reflects surface protein composition rather than differential EV capture on the sensor surface, a sandwich immunofluorescence assay was performed on the IME biosensor after impedance measurement (**Figure S7H**). Following EV immobilization and impedance acquisition, Alexa 488-conjugated anti-CD63 monoclonal antibody (MEM-259 clone), diluted 1:200 in 1× PBS, was applied and incubated for 2 h. After washing with 1× PBS and deionized water to remove residual antibody, fluorescence images were acquired. A region of interest (ROI) of 100 × 150 μm was defined at the center of the sensing area, and green channel fluorescence was isolated using ImageJ to obtain the mean intensity, which served as an indicator of the quantity of surface-immobilized EVs. Concentration-dependent analysis revealed weak semi-logarithmic trends in fluorescence intensity for both EV types, confirming that EV capture increased with concentration (**Figure S7I**). Importantly, at 10^5^ particles mL^-1^, the concentration used for the group comparison in **Figure S7E–G**, fluorescence intensities were comparable between UEVs and MEVs (p = 0.94; **Figure S7J**), indicating that the amount of captured EVs did not differ significantly between the two groups. This result suggests that the impedance differences, particularly in reactance, are attributable to differences in surface protein abundance rather than differential EV capture efficiency on the sensor surface.

**References**

[1]	H. Lee, S. Kim, D. Kong, H. Park, H.-J. Park, J. Kim, *Advanced Materials Technologies* ***2026***, e02310.
